# Efficacy and safety of baricitinib and tocilizumab in hospitalized patients with COVID-19: A comparison using systematic review and meta-analysis

**DOI:** 10.3389/fphar.2022.1004308

**Published:** 2022-10-14

**Authors:** Jerin Jose Cherian, Madhavi Eerike, Bhavani Shankara Bagepally, Saibal Das, Samiran Panda

**Affiliations:** ^1^ Indian Council of Medical Research, New Delhi, India; ^2^ Department of Pharmacology, All India Institute of Medical Sciences, Bibinagar, India; ^3^ Indian Council of Medical Research – National Institute of Epidemiology, Chennai, India; ^4^ Indian Council of Medical Research – Centre for Ageing and Mental Health, Kolkata, India; ^5^ Department of Global Public Health, Karolinska Institutet, Stockholm, Sweden

**Keywords:** coronavirus disease 2019 (COVID-19), immunomodulators, interleukin-6 inhibitors, JAK-STAT inhibitors, 28-day mortality

## Abstract

**Objective:** This review was performed to compare the efficacy and safety among hospitalized patients with COVID-19 who received baricitinib and those who received tocilizumab independently with placebo or the standard of care (SOC).

**Methods:** Relevant databases were searched for randomized controlled trials which evaluated the effect of baricitinib or tocilizumab as compared to placebo or the SOC in hospitalized patients with COVID-19. The primary endpoint was the comparison of the 28-day mortality. Risk ratios (RR) and mean differences were compared and pooled for dichotomous and continuous variables, respectively. A two-staged exploratory network meta-analysis using a multivariate meta-analysis was also performed. All analyses were performed in Stata version 16.0. The GRADE approach was used to assess the quality of the generated evidence (PROSPERO ID: CRD42022323363).

**Results:** Treatment with baricitinib [RR, 0.69 (95% CI, 0.50–0.94), *p* = 0.02, i^2^ = 64.86%] but not with tocilizumab [RR, 0.87 (95% CI, 0.71–1.07), *p* = 0.19, i^2^ = 24.41%] led to a significant improvement in the 28-day mortality as compared to that with the SOC. Treatment with baricitinib or tocilizumab, both independently led to a significant reduction in the duration of hospitalization [baricitinib: mean difference, −1.13 days (95% CI, −1.51 to −0.76), *p* < 0.001, i^2^ = 0.00%; tocilizumab: mean difference, −2.80 days (95% CI, −4.17 to −1.43), *p* < 0.001, i^2^ = 55.47%] and a significant improvement in the proportion of patients recovering clinically by day 28 [baricitinib: RR, 1.24 (95% CI, 1.03–1.48), *p* = 0.02, i^2^ = 27.20%; tocilizumab: RR, 1.41 (95% CI, 1.12–1.78), *p* < 0.001, i^2^ = 34.59%] as compared to those with the SOC. From the safety point of view, both these drugs showed similar results. There were fewer patients who experienced any serious adverse event following treatment with barictinib and tocilizumab as compared to those following treatment with the SOC [baricitinib: RR, 0.76 (95% CI, 0.62–0.92), *p* = 0.01, i^2^ = 12.63%; tocilizumab: RR, 0.85 (95% CI, 0.72–1.01), *p* = 0.07, i^2^ = 0.00%].

**Conclusion:** As baricitinib and tocilizumab are recommended interchangeably by various guidelines for the management of COVID-19, considering the better 28-day mortality data and other comparable efficacy and safety outcomes, baricitinib may be favored over tocilizumab considering its ease of administration, shorter half-life, and lower cost of treatment.

## Introduction

Coronavirus Disease 2019 (COVID-19) was declared a Public Health Emergency of International Concern by the World Health Organization (Statement on the second meeting of the International Health Regulations (2005) Emergency Committee regarding the outbreak of novel coronavirus (2019-nCoV), 2020). COVID-19 has so far resulted in >544 million cases and >6.3 million deaths globally (WHO Coronavirus Dashboard, 2022). There is also evidence of a significant burden in terms of disability-adjusted life years lost (Fan et al., 2021), morbidity (Fan et al., 2021), and poor mental wellbeing (Groff et al., 2021; Beckstein et al., 2022).

Dysregulation of the immune system in patients affected with COVID-19 is associated with poorer outcomes. In the post-viremic phase, elevated levels of inflammatory markers, including C-reactive protein, ferritin, interleukin (IL)-1, and IL-6 mark the immune origin of the worsening of respiratory symptoms (Que et al., 2022). Besides antiviral agents, immunomodulation is therefore considered an adjunct therapeutic component for the management of immune hyperactivation in moderate-to-severe COVID-19 (van de Veerdonk et al., 2022). Corticosteroids (Thakur, Datusalia and Kumar, 2022) and various immunomodulators that have some role in the management of COVID-19 include Janus-kinase signal transducers and activators of transcription (JAK-STAT) inhibitors, IL-6 inhibitors, and IL-1 receptor blockers (Ngamprasertchai et al., 2022).

JAK-STAT inhibitors, such as baricitinib, tofacitinib, and ruxolitinib, are used in patients with COVID-19 since they interfere with the inflammatory signal transduction process (Limen et al., 2022). Similarly, IL-6 inhibitors, such as tocilizumab and sarilumab have shown mortality benefits in patients with inflammatory decompensation and are believed to modulate the inflammatory cascade (Rubin et al., 2021). Various guidelines on the management of COVID-19 (Agarwal et al., 2020; NICE 2022; Adults, Hospitalized Adults: Therapeutic Management, 2022; AIIMS/ICMR-COVID-19, 2022) recommend the use of tocilizumab in patients with severe or rapidly progressing COVID-19 who are on corticosteroid treatment. In similar patients, baricitinib has also been recommended as an alternative to IL-6 inhibitors (Agarwal et al., 2020; NICE 2022; Adults, Hospitalized Adults: Therapeutic Management, 2022). The quantum and strength of evidence are strongest for baricitinib among the different JAK-STAT inhibitors (Zhang X. et al., 2022) and for tocilizumab among the various IL-6 inhibitors (WHO Rapid Evidence Appraisal for COVID-19 Therapies (REACT) Working Group et al., 2021). These two immunomodulators are used almost interchangeably. Retrospective cohort studies have shown that hospitalized patients who received baricitinib or tocilizumab have similar efficacy (survival) and safety outcomes (Kojima et al., 2022; Roddy et al., 2022).

However, there are no direct comparisons in any trials between baricitinib and tocilizumab, and hence, it is difficult to recommend one drug over the other. The guidelines urge clinicians to consider factors, including local guidance, ease of administration, access, storage, and cost of treatment (Agarwal et al., 2020; NICE 2022; Adults, Hospitalized Adults: Therapeutic Management, 2022). Further, the inconsistency in the 28-day mortality benefit of tocilizumab across various studies, makes it hard to conclusively estimate its efficacy (Agarwal et al., 2020; Gupta et al., 2022). On the other hand, trials with baricitinib have consistently reported a 28-day mortality benefit (Selvaraj et al., 2022). This makes it all the more important to explore the benefits of baricitinib over tocilizumab through indirect comparison. Hence, we aimed to compare the efficacy and safety among hospitalized patients with COVID-19 who received either one of these, independently with the standard of care (SOC). Further, we intended to exploratively assess the indirect comparison using a network meta-analysis of the efficacy and safety of these two drugs. Such information may aid clinicians in clinical decision-making while managing patients admitted with COVID-19.

## Materials and methods

### Study design

Randomized controlled trials that evaluated the effect of baricitinib or tocilizumab as compared to placebo or the SOC in hospitalized patients clinically diagnosed with COVID-19 were included. Trials that included baricitinib in combination with tocilizumab, those which included JAK-STAT inhibitors without baricitinib (e.g. ruxolitinib, tofacitinib, *etc.*), those which included IL-6 Inhibitors without tocilizumab (e.g. sarilumab, siltuximab, *etc.*), and those which evaluated any other concomitant immune-modulator were excluded. Articles with full-text access were only considered; while conference proceedings, review articles, commentaries, *etc.* were excluded. The primary outcome of this study was the comparison of the 28-day mortality (Dodd et al., 2020). The secondary outcomes were the comparisons of the 14-day mortality, the duration of hospitalization, the proportion of patients requiring mechanical ventilation (MV) by day 28, the proportion of patients requiring intensive care unit (ICU) admission by day 28, the duration of ICU stay by day 28, the duration of MV by day 28, the duration of ventilator-free days by day 28, the proportion of patients recovering clinically by day 28, and the proportion of patients experiencing serious adverse events (SAEs) (including serious infections, cardiac SAEs, venous thromboembolic events, serious bleeding episodes, and undergoing treatment discontinuation) (Dodd et al., 2020).

### Search strategy

PubMed, Embase, Scopus, Cochrane CENTRAL, medRxiv (pre-print), bioRxiv (pre-print), and ClinicalTrials.gov were searched for studies published between 1 December 2019 and 31 March 2022. The search strategy is provided in Supplementary Table S1. For various bibliographic databases, the search terms were adapted along with database-specific filters. The titles and abstracts of relevant studies in English were found out using the search strategy by two independent authors. To determine the suitability, these authors then retrieved the study abstracts, and if necessary, the full text of the articles. The web-based Rayyan software (https://www.rayyan.ai/) was used for this purpose. For accessing the missing information, the corresponding authors of the relevant article were contacted by email.

### Data extraction and management

Two authors independently extracted data using a standardized data extraction spreadsheet. The extracted data included the general characteristics of the articles, population, intervention, comparison group, and outcome of interest (according to the study objectives) for pooling. During data extraction, no simplifications or assumptions were made. Data of the intention-to-treat analysis only were evaluated. Any attrition, such as loss to withdrawals, follow-up, and dropouts were investigated. Other issues of missing data and data imputation were critically appraised (Higgins et al., 2019). For the assessment of the risk of bias in various studies, the revised Cochrane risk-of-bias 2 tool for randomized controlled trials was used (Sterne et al., 2019). Two authors independently validated the assessment, and discrepancies or disagreements were resolved in consultation with a third author. The efficacy outcomes between baricitinib and tocilizumab with the SOC were directly compared and pooled if three or more studies contained the data of interest. For insufficient data, descriptive statistics were used. For dichotomous variables, the risk ratio (RR) was calculated for each study and then pooled across studies using a random-effect model (i.e., DerSimonian-Laird). Likewise, for continuous variables, the mean differences [with 95% confidence intervals (CI)] were pooled using a random-effect model (i.e., DerSimonian-Laird).

Heterogeneity was explored using forest plot, the Cochrane Q test, and i^2^ statistics (Higgins et al., 2003). The heterogeneity of treatment effects was considered present if the *p*-value from the Cochrane Q test was <0.10 or i^2^ was >25%. If heterogeneity was present, the source of heterogeneity was explored by assessing whether clinical or methodological factors were associated with the effect sizes using a meta-regression analysis. The demographic and clinical characteristics, as well as the methodological factors, were fitted one by one in the meta-regression model. If any particular factor could explain the variation of the treatment effect, i.e., a regression coefficient was significant or tau-squared had decreased by >50%, then that factor was considered as the source of heterogeneity.

For network meta-analysis, the parameters and methods for pooling data were the same as those for the direct meta-analysis. An exploratory network meta-analysis was performed to assess the treatment effects between the interventions. The interventions SOC, baricitinib, and tocilizumab were coded as 1, 2, and 3, respectively, with SOC as the reference or the common comparator. A network of these three interventions was mapped consisting of nodes of edges. The nodes were weighed by the number of included studies in each treatment comparison, while the edges were weighed by the sample size of that comparison. A contribution plot was developed to display the contribution of each direct comparison to the network meta-analysis estimation. A two-staged approach was used for this network meta-analysis using a multivariate meta-analysis. In the first stage, a binary regression analysis was performed to estimate relative treatment effects in each study along with variance-covariance for each study by setting SOC as the reference group. In the second stage, a network meta-analysis was performed using a multivariate random-effect meta-analysis with a consistency model to pool the effect size across the studies. Based on this network meta-analysis model, multiple relative treatment comparisons were made. The treatment was ranked the best if the RR was the lowest (indicators: rankogram and surface under the cumulative ranking curve). Since no connected network was found, consistency was assumed. The transitivity and heterogeneity in the network were not explored in detail. Publication bias was not assessed considering the limited number of studies available.

All the statistical analyses were performed using Stata software version 16.0 (Stata Corp, TX, United States). A *p*-value (two-sided) of <0.05 was considered statistically significant for all tests, except for heterogeneity for which a one-sided *p*-value of <0.1 was considered statistically significant. Finally, the GRADE approach (Grading of Recommendations Assessment, Development and. Evaluation) was used to assess the quality of generated evidence for the outcomes for which pooled analyses were performed (Andrews J. C. et al., 2013; Andrews J. et al., 2013). The study protocol can be accessed in PROSPERO (ID: CRD42022323363).

## Results

**FIGURE 1:**
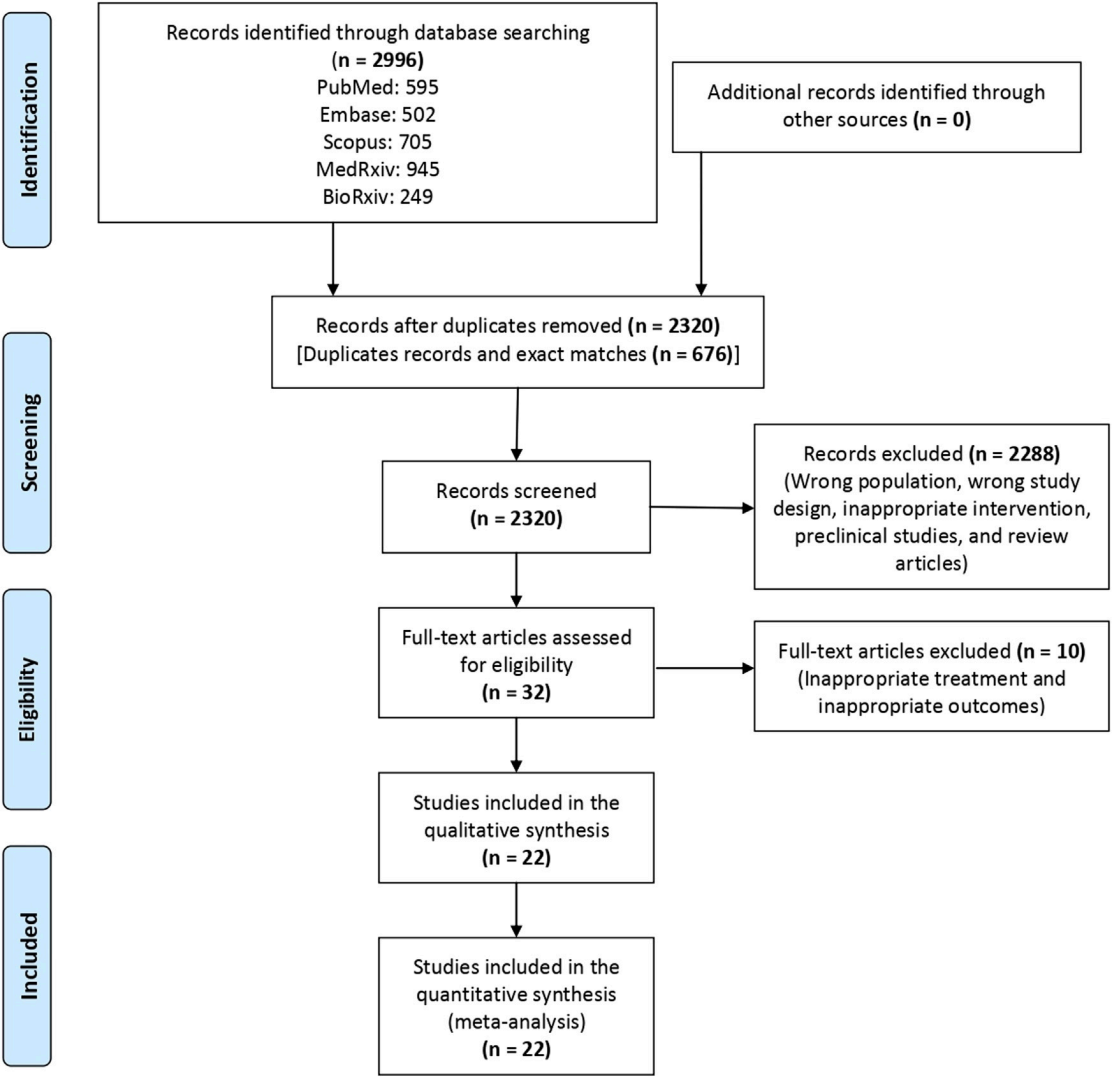
Study flow chart depicting the steps of the synthesis of evidence from the literature.

A total of 2,995 articles were screened, and finally, 22 articles (Stone et al., 2020; Kalil et al., 2021; Abani et al., 2021; Marconi et al., 2021; Declercq et al., 2021; REMAP-CAP Investigators et al., 2021; Hermine et al., 2021; Naik et al., 2021; Rosas, et al., 2021a; Rosas et al., 2021b; Rutgers et al., 2021; Salama and Mohan, 2021; Veiga et al., 2021; Wang et al., 2021; Zhao et al., 2021; Salvarani et al., 2021; Soin et al., 2021; Ely et al., 2022; Broman et al., 2022; RECOVERY Collaborative Group, 2022; Hermine, et al., 2022a; Hermine et al., 2022b) were included in the review (Figure 1). Of these 22 articles, four (Kalil et al., 2021; Marconi et al., 2021; Ely et al., 2022; RECOVERY Collaborative Group, 2022) evaluated the efficacy of baricitinib and 18 (Stone et al., 2020; Abani et al., 2021; Rosas et al., 2021a; Rosas, et al., 2021b; Declercq et al., 2021; Hermine et al., 2021; Naik et al., 2021; REMAP-CAP Investigators et al., 2021; Rutgers et al., 2021; Salama and Mohan, 2021; Salvarani et al., 2021; Soin et al., 2021; Veiga et al., 2021; Wang et al., 2021; Zhao et al., 2021; Hermine et al., 2022a; Hermine et al., 2022b; Broman et al., 2022) evaluated the efficacy of tocilizumab as compared to the SOC in hospitalized patients with COVID-19. The characteristics of the individual studies that were included for evidence synthesis are enumerated in Table 1. The result of the risk of bias assessment following the revised Cochrane risk-of-bias 2 tool is depicted in Supplementary Figure S1. Half of the included studies had a moderate risk of bias.

**TABLE 1:** Characteristics of the individual studies that were included for evidence synthesis.

Author, year	Countries where the trial was conducted	Diagnostic tool for COVID-19	Age (years)^a^	Number of patients (male: female ratio)	Primary outcome	Key inclusion criteria	Key exclusion criteria	Interval between symptom onset or diagnosis and enrolment (days)	Interventional drug	Dose, frequency, and duration of interventional drug	Comparator drug	Standard of care^b^	Duration of follow-up (days)
Clinical trials with baricitinib													
RECOVERY Collaborative Group, (2022)	UK	Clinically suspected or laboratory-confirmed cases	B arm: 58.5 ± 15.4, C arm: 57.7 ± 15.5	B arm: 4148 (66: 34), C arm: 4008 (66: 34)	Mortality by day 28	Age ≥2 years	Age of <2 years, eGFR of <15 ml/min/1.73 m^2^ or on dialysis or hemofiltration, neutrophil count of <0.5 × 10^9^ 116/L, active tuberculosis, and pregnancy or lactation	B arm: 9 (6–12), C arm: 9 (6–11)	Baricitinib	4 mg/day orally daily for 10 days or until discharge	Placebo	Cortecosteroids, remdesivir, casirivimab + imdevimab, and tocilizumab	28
Kalil et al. (2021)	US, Singapore, South Korea, Mexico, Japan, Spain, UK, and Denmark	RT-PCR	B arm: 55 ± 15.4, C arm: 55.8 ± 16	B arm: 515 (61.9: 38.1), C arm: 518 (64.3: 35.7)	Time to recovery by day 28	Age ≥18 years and lower respiratory tract infection	ALT or AST level of >5-times the upper limit of normal, impaired renal function, need for hemodialysis or hemofiltration, hypersensitivity to study drugs, pregnancy or lactation, and anticipated discharge or transfer to another hospital within 72 h	B arm: 8 (5–10), C arm: 8 (5–11)	Baricitinib + remdesivir	4 mg/day orally or through a nasogastric tube daily for 14 days or until discharge	Placebo + remdesivir	Not mentioned	29
Marconi et al. (2021)	Germany, Italy, Spain, UK, US, Argentina, Brazil, India, Japan, South Korea, Mexico, and Russia	RT-PCR or other commercial or public health assay	B arm: 57.8 ± 14.3, C arm: 57.5 ± 13.8	B arm: 764 (64: 36), C arm: 761 (62: 38)	Proportion of patients progressing to high-flow O_2_ or NIV, MV or ECMO, or death by day 28	Age ≥18 years, hospitalized, pneumonia or active and symptomatic disease, and at least one elevated inflammatory marker	Invasive MV, receiving immunosuppressant, ever receipt of convalescent plasma or intravenous immunoglobulin, neutropenia, lymphopenia, ALT or AST level of >5-times the upper limit of normal, or eGFR of <30 ml/min/1.73 m^2^	—	Baricitinib	4 mg/day orally (2 mg/day if eGFR is 30 to <60 ml/min/1.73 m^2^) daily for 14 days or until discharge	Placebo	Systemic corticosteroids and antivirals (remdesivir)	60
Ely et al. (2022)	Argentina, Brazil, Mexico, and US	RT-PCR or other commercial or public health assay	B arm: 58.4 ± 12.4, C arm: 58.8 ± 15.2	B arm: 51 (49: 51), C arm: 50 (60: 40)	Exploratory trial: no primary outcome (pre-specified key endpoints: all-cause mortality at day 28 and day 60; number of ventilator-free days; overall improvement on NIAID-OS on days 4, 7, 10, 14, and 28; proportion of participants with at least 1-point improvement on the NIAID-OS or live discharge from hospital on days 4, 7, 10, 14, and 28; duration of hospitalization; and time to recovery through day 28)	Age ≥18 years, hospitalized, use of invasive MV or ECMO, pneumonia, and progression risk with at least one elevated inflammatory marker	Receiving high-dose corticosteroids, immunosuppressant, biologics, T-cell or B-cell-targeted therapies, interferon, or Janus-kinase inhibitors; those who received convalescent plasma or intravenous immunoglobulin; and had suspected serious active bacterial, fungal, or other infection, or untreated tuberculosis	B arm: 4 (2–7), C arm: 4 (2–7)	Baricitinib	4 mg/day orally 2 mg/day if eGFR is 30 to <60 ml/min/1.73 m^2^ daily for 14 days or until discharge	Placebo	Corticosteroids, anticoagulants, antivirals, and vasopressors	60
Clinical trials with tocilizumab													
Abani et al. (2021)	UK	Clinically suspected or laboratory-confirmed cases	T arm: 63.3 ± 13.7, C arm: 63.9 ± 13.6	T arm: 2022 (66: 34), C arm: 2094 (69: 31)	Mortality by day 28	Age ≥18 years	Hypersensitivity to tocilizumab, active tuberculosis, and evidence of active bacterial, fungal, viral, or other infection	T arm: 9 (7–13), C arm: 10 (7–14)	Tocilizumab	800 mg i.v. if bodyweight >90 kg, 600 mg if bodyweight >65 and ≤90 kg, 400 mg if bodyweight >40 and ≤65 kg, and 8 mg/kg if bodyweight ≤40 kg (additional dose if there was no clinical improvement in 12–24 h)	SOC	Corticosteroids, remdesivir, and anticoagulants	28
Broman et al. (2022)	Finland	Laboratory confirmed cases	T arm: 58.4 ± 14.1, C arm: 58.8 ± 13.7	T arm: 57 (59.6: 40.1), C arm: 29 (48.3: 51.7)	Clinical status at day 28 on an ordinal scale	Age ≥18 years, hospitalized, SPO_2_ of 93%, respiratory rate of >30/min, and at least two elevated inflammatory markers	Hypersensitivity to monoclonal antibody, active co-infection, imminent death within 24 h, on long-term immunosuppressant, pregnancy or lactation, neutrophil count of ≤1 × 10^9^/L, and platelet count of <50 × 10^3^/µl	T arm: 10 (4–18), C arm: 10 (4–18)	Tocilizumab	400 mg i.v. if bodyweight <60 kg, 600 mg if bodyweight 60–90 kg, and 800 mg if bodyweight >90 kg	SOC	Low-molecular-weight heparin and corticosteroids (no antivirals, hydroxychloroquine, or experimental drugs)	28
Declercq et al. (2021)	Belgium	Laboratory confirmed cases	T arm: 65 (54–73), C arm: 64 (55–72)	T arm: 155 (77: 23), C arm: 115 (78: 22)	Time to clinical improvement or to discharge alive	Age ≥18 years, symptoms between 6 and 16 days, PaO_2_: FiO_2_ ratio of <350 mm Hg on room air or <280 mm Hg on supplemental O_2_ and bilateral pulmonary infiltrates, and cytokine release	MV for >24 h, clinical frailty score of >3 before COVID-19, unlikelihood to survive beyond 48 h, active co-infection, thrombocytopenia, neutropenia, history of bowel perforation or diverticulitis, or high dose systemic corticosteroid or immunosuppressant use for COVID-19-unrelated disorders	T arm: 10 (8–12), C arm: 10 (9–12)	Tocilizumab	8 mg/kg i.v. single dose	SOC	Hydroxychloroquine, and dexamethasone	28
REMAP-CAP Investigators et al. (2021)	Canada, US, France, Germany, Ireland, Netherlands, Portugal, UK, Saudi Arabia, Australia, and New Zealand	Clinically suspected or RT-PCR	T arm: 61.5 ± 12.5, C arm: 61.1 ± 12.8	T arm: 353 (74: 26), C arm: 402 (70: 30)	Number of respiratory and cardiovascular organ support–free days up to day 21	Age ≥18 years, critically ill, and admitted to ICU and receiving respiratory or cardiovascular support	Imminent death with lack of commitment to full support or if they had previously participated in the REMAP-CAP trial within 90 days	—	Tocilizumab	8 mg/kg i.v. single dose (additional 6 mg/kg if there was no clinical improvement in 12–24 h)	SOC	Corticosteroids	90
Hermine et al. (2021)	France	RT-PCR or CT scan	T arm: 64 (57.1–74.3), C arm: 63.3 (57.1–72.3)	T arm: 63 (70: 30), C arm: 67 (66: 34)	Proportion of patients dead or needing non-invasive or MV on day 4 and survival with no need for non-invasive or MV by day 14	Moderate or severe pneumonia requiring at least 3 L/min of O_2_ but without MV or admission to the ICU	Hypersensitivity to tocilizumab, pregnancy, current bacterial infection, and absolute neutrophil count of ≤1 × 10^9^/L or platelet count of <50 × 10^3^/µl	T arm: 10 (7–13), C arm: 10 (8–13)	Tocilizumab	8 mg/kg i.v. single dose (additional 400 mg on day 3 if O_2_ requirement was not decreased by >50%)	SOC	Antibiotics, antivirals, corticosteroids, vasopressors, and anticoagulants	90
Hermine, et al. (2022a)	France	RT-PCR or CT scan	T arm: 63.2 (59.4–70.9), C arm: 65.4 (57.6–70.5)	T arm: 49 (67: 33), C arm: 43 (77: 23)	Early: proportion of patients with a decrease of WHO score of at least 1 point at day 4. Longer: cumulative incidence of successful tracheal extubation at day 14 or removal of NIV or high flow O_2_	Adult; moderate, severe, or critical pneumonia (O_2_ requirement of >3 L/min); and WHO Clinical Progression Scale score of >5, including patients on NIV or MV	Hypersensitivity to tocilizumab, pregnancy, bacterial infection, absolute neutrophil count of ≤1 × 10^9^/L, and platelet count of <50 × 10^3^/µl	T arm: 11 (9–15), C arm: 11 (9–14)	Tocilizumab	8 mg/kg i.v. single dose (additional 400 mg on day 3 if O_2_ requirement was not decreased by >50%)	SOC	Antibiotics, antivirals, corticosteroids, vasopressors, and anticoagulants	90
Hermine, et al. (2022b)	France	RT-PCR or CT scan	T arm: 63.6 (52.6–73.3), C arm: 63.2 (53.6–73.3)	T arm: 224 (65: 35), C arm: 226 (70: 30)	Survival without invasive ventilation at day 14	Adult; moderate and severe pneumopathy requiring O_2_ (>3 L/min) but without NIV, high flow O2, NIV, or MV; and WHO Clinical Progression Scale score of 5	Hypersensitivity to tocilizumab, pregnancy, bacterial infection, absolute neutrophil count of ≤1×10^9^/L, platelet count of <50 × 10^3^/µl, ALT level of ≥5-times the upper limit of normal	T arm: 9 (7–11), C arm: 9 (7–11)	Tocilizumab	8 mg/kg i.v. single dose (additional 400 mg on day 3 if O_2_ requirement was not decreased by >50%)	Dexamethasone (10 mg/day for 5 days and tapering up to 10 days)	Antibiotics, antivirals, vasopressors, anticoagulants, renal-replacement therapy, and ECMO	90
Naik et al. (2021)	India	RT-PCR	T arm: 50 (44–65), C arm: 51 (45–58)	T arm: 21 (57.1: 42.9), C arm: 21 (57.1: 42.9)	Ventilator-free days till day 28	Age ≥18 years, PaO2: FiO2 ratio of <200 on admission and those with clinical worsening in <48 h of initiation of SOC	History of immunosuppressant use, invasive bacterial or fungal infection, ALT or AST level of ≥5-times the upper limit of normal, leukocyte count of <2 × 10^3^/μl, platelet count of <50 × 10^3^/μl, and acute or chronic diverticulitis	T arm: 8 (7–9), C arm: 7 (7–8)	Tocilizumab	8 mg/kg i.v. single dose (additional 6 mg/kg if there was no clinical improvement within 24 h)	Dexamethasone (20 mg i.v./day for 3 days)	Remdesivir, and low-molecular-weight heparin	28
Rosas, et al. (2021a)	Canada, Denmark, France, Germany, Italy, Netherlands, Spain, UK, and US	RT-PCR and chest radiograph or CT scan	T arm: 60.9 ± 14.6, C arm: 60.6 ± 13.7	T arm: 294 (69.7: 30.3), C arm: 144 (70.1: 29.9)	Clinical status at day 28 on an ordinal scale	Age ≥18 years, with severe pneumonia, SPO_2_ of 93%, and PaO2: FiO2 ratio of <300 mm Hg	Imminent or inevitable death within 24 h, active tuberculosis, bacterial, fungal, or viral infection	T arm: 12.1 ± 6.6, C arm: 11.4 ± 6.9	Tocilizumab	8 mg/kg i.v. single dose	Placebo	Antivirals, corticosteroids, convalescent, and plasma	28
Rosas, et al. (2021b)	Brazil, Russia, Spain, and US	RT-PCR	T arm: 60.1 ± 13.3, C arm: 58.2 ± 13.3	T arm: 430 (61.9: 38.1), C arm: 210 (66.2: 33.8)	Time from randomization to hospital discharge or ready for discharge till day 28	Pneumonia and hypoxemia requiring >6 L/min O_2_	eGFR of <30 ml/min/1.73 m^2^, ALT or AST level of >5-times the upper limit of normal, and bacterial, fungal, or viral infection	T arm: 8.8 ± 4.8, C arm: 8.9 ± 4.7	Tocilizumab + remdesivir	8 mg/kg i.v. single dose (additional 6 mg/kg if there was sustained fever or clinically significant worsening of signs and symptoms)	Placebo + remdesivir	Azithromycin and corticosteroids	28
Rutgers, et al. (2021)	Netherlands	RT-PCR	T arm: 67 (60–74), C arm: 66 (56–75)	T arm: 174 (67: 33), C arm: 180 (67: 33)	Mortality by day 30	Age ≥18 years and hyper-inflammation	Not mentioned	T arm: 8 (5–10), C arm: 8 (6–10)	Tocilizumab	8 mg/kg i.v. single dose (additional 6 mg/kg if there was hypoxia after 8 h)	SOC	Dexamethasone, hydroxychloroquine, and remdesivir	30
Salama and Mohan, (2021)	Brazil, Kenya, Mexico, Peru, South Africa, and US	RT-PCR and chest radiograph	T arm: 56 ± 14.3, C arm: 55.6 ± 14.9	T arm: 249 (60.2: 39.8), C arm: 128 (57: 43)	MV or death by day 28	Age ≥18 years and SpO_2_ of <94% in ambient air	Continuous positive airway pressure, bi-level positive airway pressure, or MV	—	Tocilizumab	8 mg/kg i.v. single dose (additional 6 mg/kg if clinical signs or symptoms worsened or did not improve)	SOC	Antivirals and corticosteroids	60
Salvarani et al. (2021)	Italy	Positive RT-PCR	T arm: 61.5 (51.5–73.5), C arm: 60 (54–69)	T arm: 60 (66.7: 33.3), C arm: 66 (56.1: 43.9)	Clinical worsening by day 14	Age ≥18 years, presence of acute respiratory failure with PaO2: FiO2 ratio of 200–300 mm/Hg, and presence of inflammatory phenotype	ICU admission, hypersensitivity to tocilizumab, any condition preventing future ICU admission, and patient’s willingness to avoid intubation	T arm: 7 (4–11), C arm: 8 (6–11)	Tocilizumab	8 mg/kg i.v. single dose (additional dose after 12 h)	SOC	Corticosteroids	30
Soin et al. (2021)	India	Positive RT-PCR	T arm: 56 (47–63), C arm: 54 (43–63)	T arm: 91 (84: 16), C arm: 88 (86: 14)	Progression of disease by day 14	Age ≥18 years and moderate to severe disease	Hypersensitivity to tocilizumab or another monoclonal antibody; active tuberculosis; and suspected or active bacterial, fungal, or viral infection (except treated hepatitis C or B)	—	Tocilizumab	6 mg/kg i.v. single dose	SOC	Hydroxychloroquine, azithromycin, remdesivir, and corticosteroids	28
Stone, et al. (2020)	US	RT-PCR or serum antibody	T arm: 61.6 (46.4–69.7), C arm: 56.5 (44.7–67.8)	T arm: 161 (60: 44), C arm: 82 (55: 45)	Intubation or death	19–85 years; at least two of the following signs: fever within 72 h, pulmonary infiltrates, or a need for supplemental O_2_ to maintain SpO_2_ of >92%; and at least one elevated inflammatory marker	Receiving O_2_ of >10 L/min, history of treatment with a biologic or small molecule, receiving other immunosuppressant, or presence of diverticulitis	T arm: 9 (6–13), C arm: 10 (7–13)	Tocilizumab	8 mg/kg i.v. single dose	Placebo	Remdesivir, hydroxychloroquine, and corticosteroids	29
Veiga et al. (2021)	Brazil	RT-PCR	T arm: 57.4 ± 15.7, C arm: 57.5 ± 13.5	T arm: 65 (68: 32), C arm: 64 (69: 31)	Clinical status at day 15 days	Severe or critical disease with evidence of pulmonary infiltrates, receiving O_2_ to maintain SpO_2_ of >93%, receiving MV for <24 h, and at least two elevated inflammatory markers	Active uncontrolled infection, ALT or AST level of >5-times the upper limit of normal, and eGFR of <30 ml/min/1.73 m^2^	T arm: 10 ± 3.1, C arm: 9.5 ± 3	Tocilizumab	8 mg/kg i.v. single dose	SOC	Hydroxychloroquine, azithromycin, corticosteroids, and antibiotics	29
Wang, et al. (2021)	China	RT-PCR	T arm: 63.5 (58–71), C arm: 63 (54–69)	T arm: 34 (52.9: 47.1), C arm: 31 (48.3: 51.7)	Cure rate	18–85 years, elevated plasma interleukin-6 level, moderate (with bilateral pulmonary lesions) or severe disease	Pregnancy or lactation; ALT or AST level of >5-times the upper limit of normal; platelet count of <50×10^3^/µl; rheumatic and immune diseases, cancer, and other related diseases; on immunosuppressant; hypersensitivity to tocilizumab; active hepatitis and tuberculosis; specific bacterial and fungal infection; history of organ transplantation; and mental disorders	T arm: 20 (9–29), C arm: 24 (19–33)	Tocilizumab	400 mg i.v. single dose (additional dose if the patient remained febrile for 24 h after the first dose)	SOC	Not mentioned	14
Zhao et al. (2021)	China	Laboratory-confirmed cases	T arm: 71 (48–77), C arm: 70 (45–89)	T arm: 5 (42.9: 57.1), C arm: 7 (60: 40)	Cumulative lung lesion remission rate	Age ≥18 years and increased interleukin-6 level	Hypersensitivity to favipiravir or tocilizumab, pregnancy or lactation; ALT or AST level of >5-times the upper limit of normal; and active hepatitis, tuberculosis, bacterial, or fungal infection	—	Tocilizumab	4–8 mg/kg i.v. single dose (additional dose if the patient remained febrile after 24 h)	Favipiravir (1,600 mg, twice daily on day 1 followed by 600 mg, twice daily on days 2–7)	Not mentioned	60

a Expressed as mean ± standard deviation or median (inter-quartile range or range).

b Supportive treatment was provided to all patients.

ALT, alanine transaminase; AST, aspartate transaminase; B arm, baricitinib; C arm, comparator arm; COVID-19, Coronavirus Disease 2019; eGFR, estimated glomerular filtration rate; ECMO, extracorporeal membrane oxygenation; FiO_2_, fraction of inspired oxygen; ICU, intensive care unit; i.v., intravenous; MV, MV; NIAID-OS, National Institute of Allergy and Infectious Disease Ordinal Scale; NIV, non-invasive ventilation; O_2_, oxygen; PaO_2_, partial pressure of oxygen; RT-PCR, reverse transcriptase-polymerase chain reaction; SOC, standard of care; T arm, tocilizumab arm; WHO, World Health Organization; UK, United Kingdom; US, United States.

The number of patients that were included in baricitinib trials was 10815 (range: 101 (Ely et al., 2022) to 8156 (RECOVERY Collaborative Group, 2022)) with a male preponderance. Barictinib was mostly compared to placebo, except in one study where remdesivir (Kalil et al., 2021) was the active comparator. The most commonly used dose of barictinib was 4 mg/day orally for 10–14 days. The duration of follow-up ranged between 28 and 60 days across the different baricitinib studies. On the other hand, the number of patients that were included in tocilizumab trials was 8504 (range: 12 (Zhao et al., 2021) to 4116 (Abani et al., 2021)) with a male preponderance. Tocilizumab was mostly compared to placebo, except in four studies where dexamethasone (Naik et al., 2021; Hermine et al., 2022b), remdesivir (Rosas, et al., 2021a), and favipiravir (Zhao et al., 2021) were the active comparators. The most commonly used dose of tocilizumab was 8 mg/kg i.v. single dose (additional dose was given after 24 h depending on the clinical condition). The duration of follow-up ranged between 14 and 90 days across different tocilizumab studies. The SOC that was most commonly used in all studies included antibiotics, antivirals, corticosteroids, vasopressors, and anticoagulants.

**FIGURE 2:**
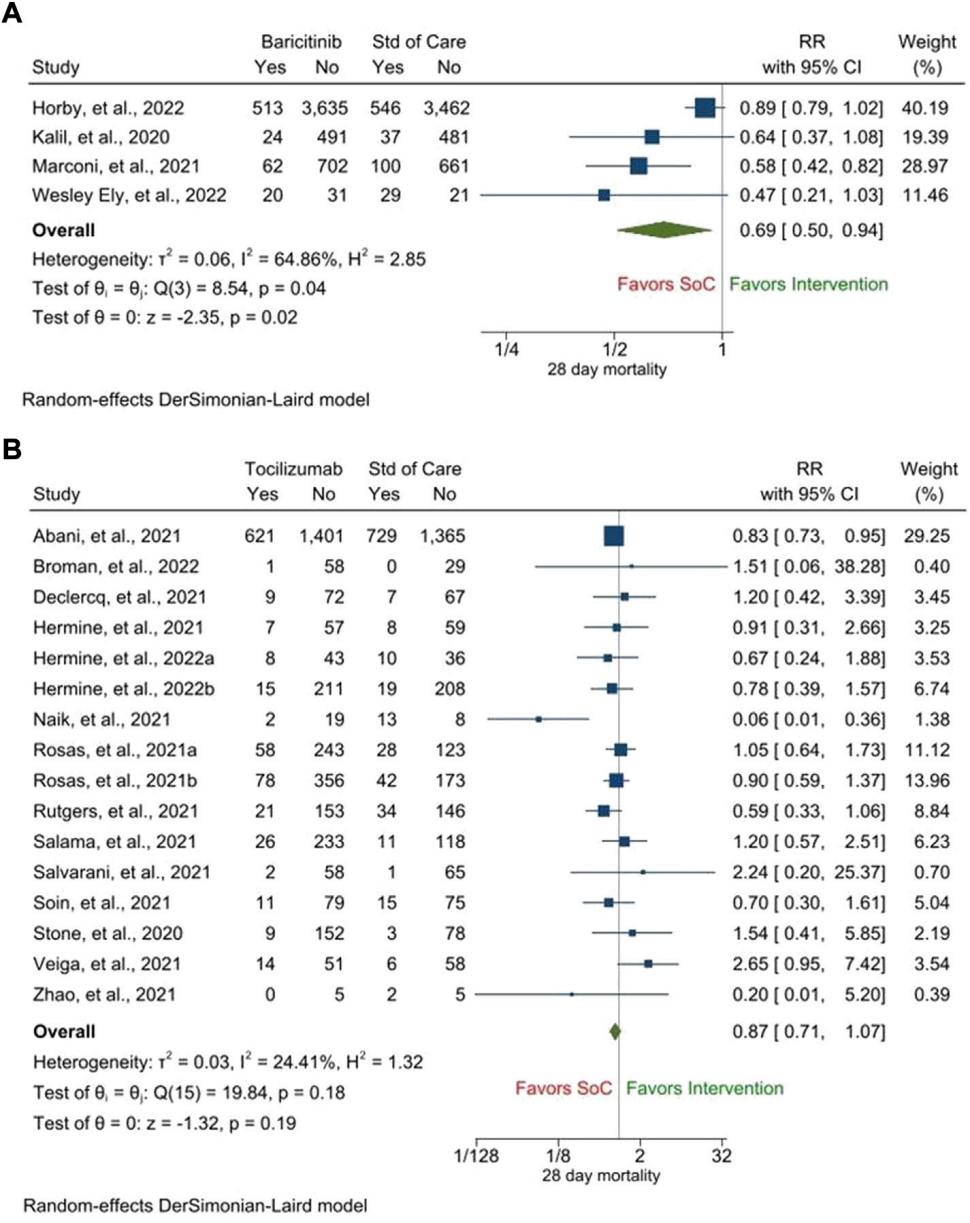
28-day mortality following treatment with baricitinib **(A)** and tocilizumab **(B)** as compared to that following treatment with the standard of care (SOC).

Regarding the primary outcome, treatment with baricitinib led to a statistically significant improvement in the 28-day mortality as compared to that with the SOC [RR, 0.69 (95% CI, 0.50–0.94), *p* = 0.02] (Figure 2A). The heterogeneity among the included studies was high (i^2^ = 64.86%). On the other hand, although treatment with tocilizumab showed a trend of improvement in the 28-day mortality as compared to that with the SOC, the improvement was not statistically significant [RR, 0.87 (95% CI, 0.71–1.07), *p* = 0.19] (Figure 2B). The heterogeneity among the included studies was low (i^2^ = 24.41%) and there was no publication bias (Supplementary Figure S2).

As far as the other secondary efficacy outcomes are concerned, only one study with baricitinib reported a 14-day mortality outcome; 8/515 patients in the baricitinib arm and 15/518 patients in the SOC arm died by 14 days (Kalil et al., 2021). On the other hand, treatment with tocilizumab did not lead to any improvement in the 14-day mortality as compared to that with the SOC [RR, 1.19 (95% CI, 0.66–2.13), *p* = 0.56, i^2^ = 30.64%] (Supplementary Figure S3). Treatment with baricitinib led to a significant reduction in the duration of hospitalization as compared to that with the SOC [mean difference, −1.13 days (95% CI, −1.51 to −0.76), *p* < 0.001, i^2^ = 0.00%] (Supplementary Figure S4). Treatment with tocilizumab also led to a significant reduction in the duration of hospitalization as compared to that with the SOC [mean difference, −2.80 days (95% CI, −4.17 to −1.43), *p* < 0.001, i^2^ = 55.47%] (Supplementary Figure S5).

Two studies with baricitinib reported the proportion of patients requiring MV by day 28. In one study, 283/4014 patients in the baricitinib arm and 322/3891 patients in the SOC arm required MV by day 28 (*p* = 0.06) (RECOVERY Collaborative Group, 2022). In the other study, 212/764 patients in the baricitinib arm and 232/761 patients in the SOC arm required MV by day 28 (*p* = 0.06) (Marconi et al., 2021). Treatment with tocilizumab led to a statistically significant improvement in the proportion of patients requiring MV by day 28 as compared to that with the SOC [RR, 0.79 (95% CI, 0.71–0.88), *p* < 0.001, i^2^ = 0.00%] (Supplementary Figure S6) and there was no publication bias (Supplementary Figure S7). No studies with baricitinib reported the proportion of patients requiring ICU admission by day 28. Treatment with tocilizumab showed a trend of reduced ICU admission by day 28 as compared to that with the SOC; however, the improvement was not statistically significant [RR, 0.83 (95% CI, 0.57–1.19), *p* = 0.30, i^2^ = 34.84%] (Supplementary Figure S8).

Only one study with baricitinib reported the duration of ICU stay by day 28; it was 3.19 ± 8.84 days in the baricitinib arm (*n* = 764) and 31.7 ± 8.54 days in the SOC arm (*n* = 761) (Marconi et al., 2021). Treatment with tocilizumab led to a reduction in the duration of ICU stay by day 28 as compared to that with the SOC; however, the reduction was not statistically significant [mean difference, −4.25 days (95% CI, −9.39 to 0.29), *p* = 0.11, i^2^ = 86.72%] (Supplementary Figure S9). Only one study with baricitinib reported the duration of MV by day 28; it was 18.94 ± 14.12 days in the baricitinib arm (*n* = 515) and 21.14 ± 12.63 days in the SOC arm (*n* = 518) (Kalil et al., 2021). On the other hand, three studies with tocilizumab reported the duration of MV by day 28. In the first study, it was 13.47 ± 6.84 days in the tocilizumab arm (*n* = 57) and 19.96 ± 15.2 days in the SOC arm (*n* = 29) (Broman et al., 2022); in the second study, it was 1.07 ± 2.38 days in the tocilizumab arm (*n* = 21) and 9.84 ± 10.33 days in the SOC arm (*n* = 21) (Naik et al., 2021); and in the third study, it was 15.0 (12.6-NR) days [median (inter-quartile range)] in the tocilizumab arm (*n* = 161) and 27.9 (16.3-NR) days [median (inter-quartile range)] in the SOC arm (*n* = 81) (Stone et al., 2020).

Two studies with baricitinib reported the duration of ventilator-free days by day 28. In one study, it was 24.5 ± 10.77 days in the baricitinib arm (*n* = 764) and 23.7 ± 10.75 days in the SOC arm (*n* = 761) (*p* = 0.05) (Marconi et al., 2021). In the other study, it was 8.1 ± 10.2 days in the baricitinib arm (*n* = 51) and 5.5 ± 8.4 days in the SOC arm (*n* = 50) (*p* = 0.21) (Ely et al., 2022). Likewise, treatment with tocilizumab did not lead to any significant reduction in the duration of ventilator-free days by day 28 as compared to that with the SOC [mean difference, 3.29 days (95% CI, −0.61–7.19), *p* = 0.10, i^2^ = 69.78%] (Supplementary Figure S10). Treatment with baricitinib led to a significant improvement in the proportion of patients recovering clinically by day 28 [RR, 1.24 (95% CI, 1.03–1.48), *p* = 0.02, i^2^ = 27.20%] (Supplementary Figure S11). Likewise, treatment with tocilizumab led to a significant improvement in the proportion of patients recovering clinically by day 28 [RR, 1.41 (95% CI, 1.12–1.78), *p* < 0.001, i^2^ = 34.59%] (Supplementary Figure S12) and there was no publication bias (Supplementary Figure S13).

**FIGURE 3:**
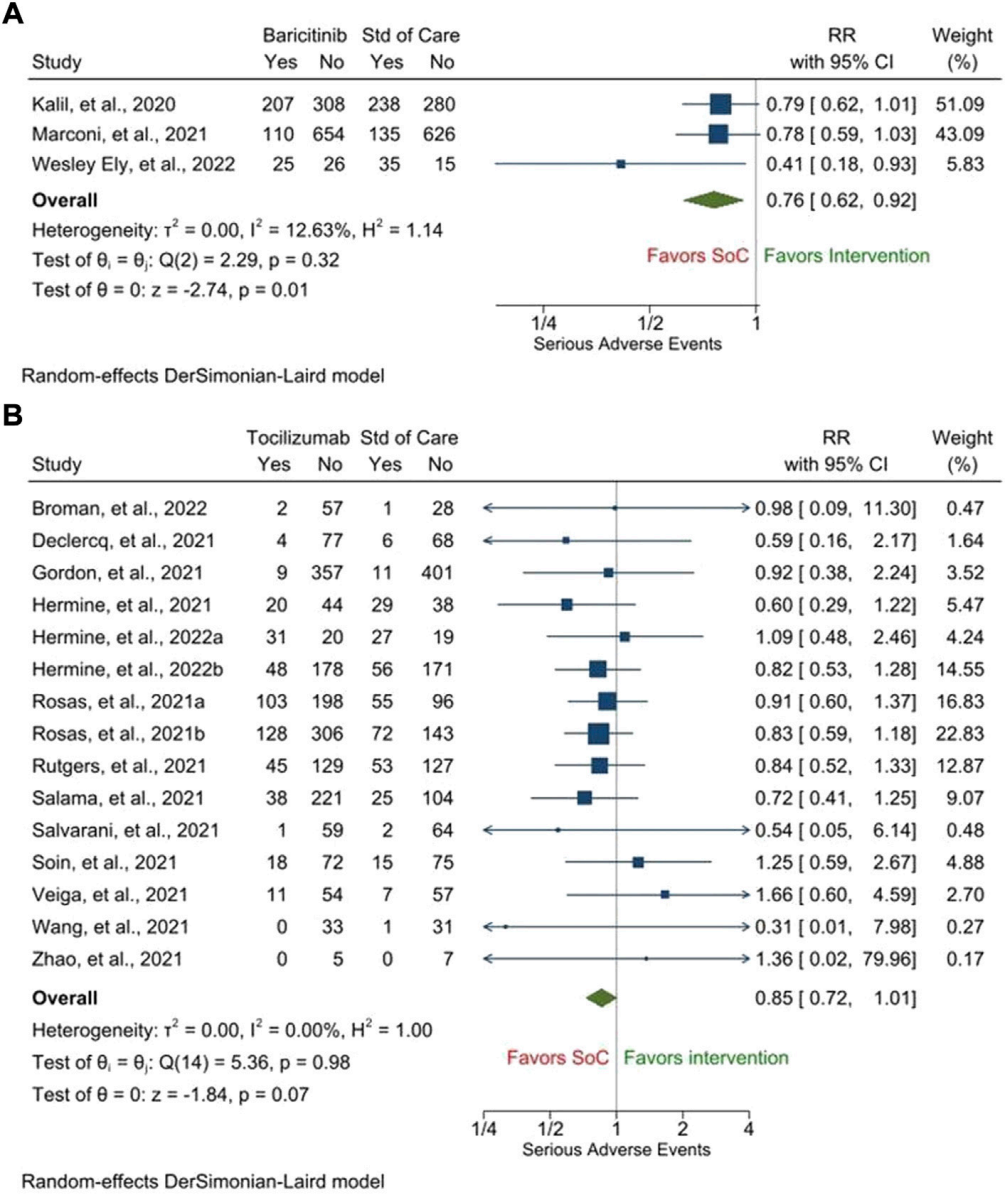
Proportion of patients experiencing any serious adverse events following treatment with baricitinib **(A)** and tocilizumab **(B)** as compared to that following treatment with the standard of care (SOC).

As far as the secondary safety outcomes are concerned, there were fewer patients who experienced any SAE [RR, 0.76 (95% CI, 0.62–0.92), *p* = 0.01, i^2^ = 12.63%] (Figure 3A], serious infections [RR, 0.86 (95% CI, 0.62–1.18), *p* = 0.34, i^2^ = 63.35%] (Supplementary Figure S14), and cardiac SAEs [0.75 (95% CI, 0.58–0.97), *p* = 0.03, i^2^ = 0.00%] (Supplementary Figure S15) following treatment with barictinib as compared to those following treatment with the SOC. However, the proportions of patients experiencing venous thromboembolism were similar between those who were treated with baricitinib and those who were treated with the SOC [RR, 1.00 (95% CI, 0.83–1.21), *p* = 0.99, i^2^ = 0.00%] (Supplementary Figure S16). Only one study with baricitinib reported the outcome of serious bleeding; 33/4148 patients in the baricitinib arm and 29/4008 patients in the SOC experienced serious bleeding (RECOVERY Collaborative Group, 2022). Two studies with baricitinib reported the proportion of patients who discontinued treatment due to SAE. In one study, it was 56/750 patients in the baricitinib arm and 70/752 patients in the SOC arm (Marconi et al., 2021). In the other study, it was 14/50 patients in the baricitinib arm and 17/49 patients in the SOC arm (Ely et al., 2022).

Likewise, there were fewer patients who experienced any SAE [RR, 0.85 (95% CI, 0.72–1.01), *p* = 0.07, i^2^ = 0.00%] (Figure 3B) [no publication bias was present (Supplementary Figure S17)], serious infections [RR, 0.68 (95% CI, 0.47–1.00), *p* = 0.05, i^2^ = 56.56%] (Supplementary Figure S18) [no publication bias was present (Supplementary Figure S19)], cardiac SAEs [0.82 (95% CI, 0.64–1.04), *p* = 0.10, i^2^ = 0.00%] (Supplementary Figure S20) [no publication bias was present (Supplementary Figure S21)], and venous thromboembolism [0.59 (95% CI, 0.22–1.55), *p* = 0.29, i^2^ = 46.88%] [Supplementary Figure S22] following treatment with tocilizumab as compared to those following treatment with the SOC. However, the proportions of patients experiencing serious bleeding were similar between those who were treated with tocilizumab and those who were treated with the SOC [RR, 1.09 (95% CI, 0.63–1.87), *p* = 0.76, i^2^ = 0.00%] (Supplementary Figure S23) [no publication bias was present (Supplementary Figure S24)]. Two studies with tocilizumab reported the proportion of patients who discontinued treatment due to SAE. In one study, it was 2/429 patients in the tocilizumab arm and 0/213 patients in the SOC arm (Rosas, et al., 2021a). In the other study, it was 0/250 patients in the tocilizumab arm and 0/127 patients in the SOC arm (Salama and Mohan, 2021). The GRADE tables representing the quality of generated evidence for the outcomes for which pooled analyses were performed are provided in Supplementary Table S2 (for baricitinib) and Supplementary Table S3 (for tocilizumab).

**FIGURE 4:**
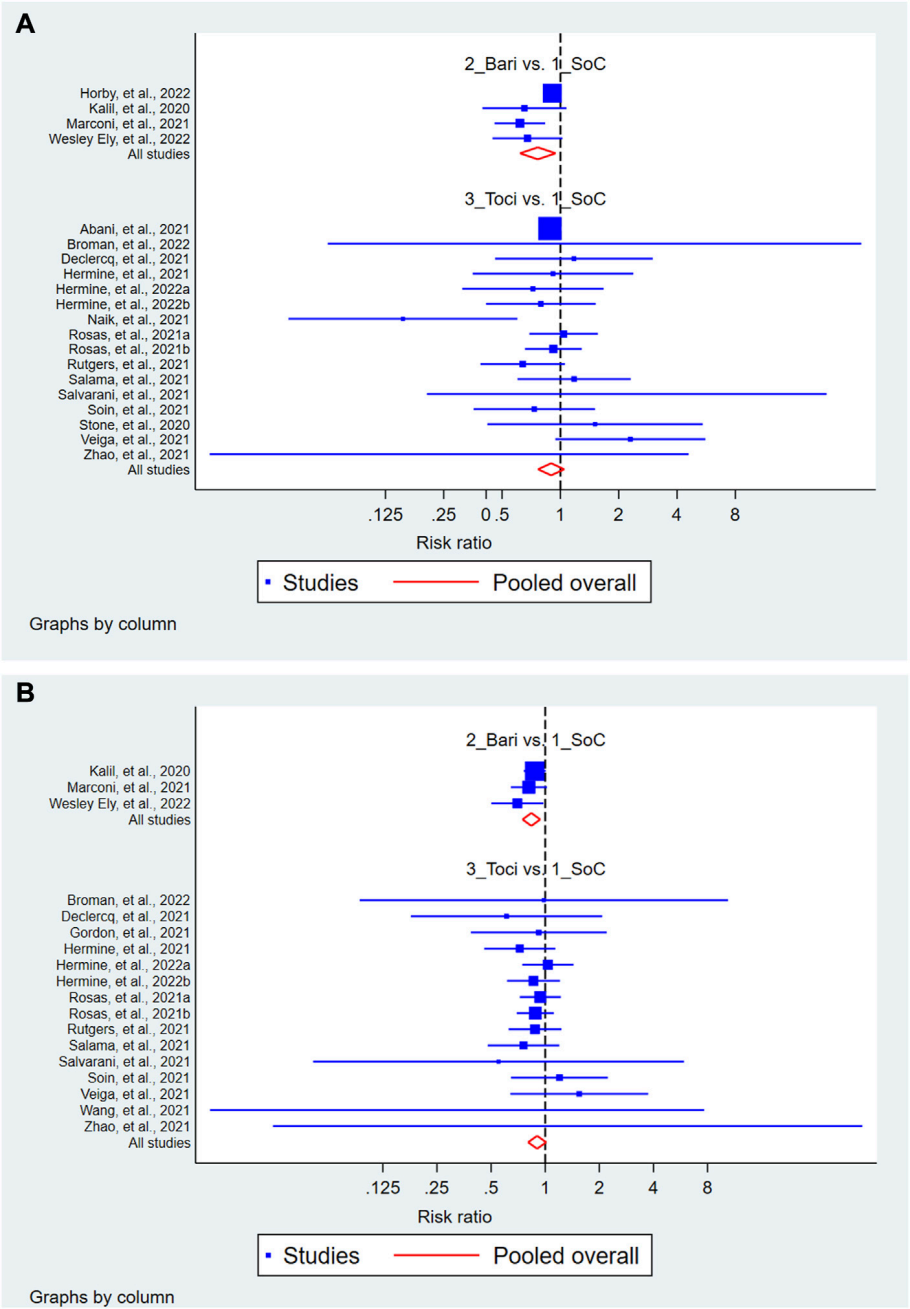
Forest of the network meta-analysis showing the 28-day mortality **(A)** and serious adverse events **(B)** following treatment with baricitinib and tocilizumab as compared to that following treatment with the standard of care (SOC).

The results of the exploratory network meta-analysis including the indirect comparison showed that treatment with baricitinib but not with tocilizumab (Figure 4A) improved the 28-day mortality as compared to that with the SOC. In the rankogram and cumulative ranking for 28-day mortality, the ranking order was baricitinib, followed by tocilizumab and SOC (Supplementary Figure S25). However, this improvement following baricitinib treatment was not sustained in the future prediction model. Similarly, treatment with baricitinib but not with tocilizumab (Figure 4B) improved the proportion of patients having SAE as compared to that with the SOC. In the rankogram and cumulative ranking, the ranking order for SAE was baricitinib, followed by tocilizumab and SOC (Supplementary Figure S26). The network maps and interval plots are illustrated in Supplementary Figures S27–S30. Also, this improvement following baricitinib treatment was sustained in the future prediction model.

## Discussion

This review was performed to compare the efficacy and safety among hospitalized patients with COVID-19 who received baricitinib and those who received tocilizumab, independently with placebo or the SOC. We found that treatment with baricitinib but not with tocilizumab led to a significant improvement in the 28-day mortality as compared to that with the SOC. There was no significant improvement in the 14-day mortality with either of these drugs. Treatment with baricitinib or tocilizumab led to a significant reduction in the duration of hospitalization and a significant improvement in the proportion of patients recovering clinically by day 28 as compared to those with SOC. Similarly, both these drugs independently showed a trend of improving the proportion of patients requiring MV by day 28, reduction in the duration of ICU stay by day 28, reduction in the duration of MV by day 28, and reduction in the duration of ventilator-free days by day 28. Further, treatment with tocilizumab showed a trend of reduction in ICU admission by day 28. From the safety point of view, both these drugs showed similar results. There was a slightly increased incidence of any SAE following treatment with barictinib or tocilizumab as compared to that following treatment with SOC. Fewer patients had serious infections and cardiac SAEs following treatment with baricitinib or tocilizumab as compared to those following treatment with the SOC. The results of venous thromboembolic events, serious bleeding episodes, and treatment discontinuation were comparable following treatment with baricitinib or tocilizumab.

Noticeably, baricitinib modulates downstream inflammatory responses *via* JAK1/JAK2 inhibition and leads to a dose-dependent inhibition of IL-6-induced STAT3 phosphorylation which is involved in vital cellular functions, including signaling, growth, and survival (McInnes et al., 2019). It exhibits antiviral effects by blocking severe acute respiratory syndrome coronavirus 2 from entering and infecting alveolar cells in the lungs (Richardson et al., 2020). Being an inhibitor of the upstream inflammatory cascade, it can reduce various cytokine levels including IL6, IL2, interferon-gamma, *etc.* (Limen et al., 2022). As the JAK-STAT signaling pathway is central to the development of cytokine storm in COVID-19, baricitinib may be useful in ameliorating it (Zhang X. et al., 2020). The anti-cytokine and anti-viral activities of baricitinib are primarily responsible for the clinical and radiological recovery, a rapid reduction in the viral load, inflammatory markers, and IL-6 levels in COVID-19 (Stebbing et al., 2020). Baricitinib has received emergency use authorization to treat COVID-19 in hospitalized adult and pediatric patients requiring supplemental oxygen, non-invasive or invasive mechanical ventilation, or extracorporeal membrane oxygenation (U.S. Food and Drug Administration, 2022).

IL-6 is a pleiotropic cytokine secreted by neutrophils, monocytes, and macrophages and involved in the inflammatory response. IL-6 promotes B and T cell differentiation, acute phase protein production, and osteoclast activation. Hyperactivation of the immune response and increased cytokine (especially IL-6) levels (Del Valle et al., 2020) lead to a compromised alveolar-capillary blood-gas exchange. This in turn causes impaired oxygen diffusion, and the ensuing inflammation eventually leads to lung fibrosis and multiorgan failure. Furthermore, elevated levels of IL-6 have been associated with a hypercoagulable state in patient with COVID-19 (Yang et al., 2020). Tocilizumab selectively and competitively binds to the IL-6 receptor and blocks IL-6-mediated signaling thereby blocking the assembling of the activated complex with the transmembrane protein (Samaee et al., 2020), mitigating immune-mediated damage, lung injury, and oxygen saturation (Zhang S. et al., 2020). In fact, it has a very specific application in patients with COVID-19 after the development of cytokine storm (Boretti and Banik, 2022). Tocilizumab has received emergency use authorization to treat COVID-19 in hospitalized adult and pediatric patients requiring systemic corticosteroids, supplemental oxygen, non-invasive or invasive mechanical ventilation, or extracorporeal membrane oxygenation (Commissioner, 2021).

It is pertinent to mention that in the absence of a head-to-head comparison between baricitinib and tocilizumab in patients with COVID-19, the only comparisons that exist between the outcomes of patients receiving these two drugs are from observational studies, which showed that their outcomes were comparable. It was also found that the initiation of baricitinib or tocilizumab among hospitalized moderate-to-severe COVID-19 patients who were on dexamethasone therapy had comparable but not significant effects on time to clinical improvement, discharge rate, recovery rate, reduction of the viral load, in-hospital mortality rate, and serious SAEs (hyperinflammatory syndrome, hepatic and renal complications, the risk of secondary infections, and thrombotic and bleeding events) (Wong et al., 2022). In hospitalized COVID-19 patients with hypoxemia and pneumonia who received dexamethasone, treatment with baricitinib or tocilizumab resulted in similar outcomes (Roddy et al., 2022).

The use of baricitinib in severe COVID-19 resulted in early stabilization of lung function, reduced requirement of critical care support, and lower re-hospitalization with mortality rates (Hasan et al., 2021). It was found that in patients with moderate to severe COVID-19, a combination of baricitinib with corticosteroids was associated with a greater improvement in lung function as compared with corticosteroids alone (Rodriguez-Garcia et al., 2021). Baricitinib administered with remdesivir and dexamethasone was shown to reduce mortality of hospitalized patients with COVID-19 (So et al., 2021). On the other hand, in a retrospective study, it was found that tocilizumab reduced mortality, although the reduction was not statistically significant, in COVID-19 patients. However, there was no increased discharge rate, risk of secondary infections, adverse events, and proportion of patients requiring MV (Jiang et al., 2021). Another study showed that tocilizumab treatment in patients with COVID-19 led to a higher extubation rate among patients who were on MV (Mohzari et al., 2022). In a different observational study, it was demonstrated that patients with COVID-19 requiring ICU support who received tocilizumab had a reduced mortality rate (Biran et al., 2020).

Similar results to ours were obtained in other systematic reviews and meta-analyses that evaluated either baricitinib or tocilizumab. A recent meta-analysis has reported that in hospitalized patients with COVID-19, baricitinib treatment led to a reduction in the 28-day mortality; however, there was no significant reduction in the proportion of patients requiring MV (Selvaraj et al., 2022). Another meta-analysis showed that baricitinib improved ICU admission, the requirement of MV, oxygenation (Lin et al., 2022), time to recovery, and mortality rate (Limen et al., 2022; Zhang X. et al., 2022). Treatment with tocilizumab was also found to reduce the 28–30-day all-cause mortality rate, ICU admission rate, risk of secondary infections, and the proportion of patients requiring MV (Avni et al., 2021). In another meta-analysis, tocilizumab treatment, as compared to the SOC or placebo, led to a lower 28-day all-cause mortality (WHO Rapid Evidence Appraisal for COVID-19 Therapies (REACT) Working Group et al., 2021). A different meta-analysis demonstrated that tocilizumab treatment could reduce the mortality rate in hospitalized COVID-19 patients and this benefit was more in patients who received concomitant corticosteroids and when tocilizumab was administered within 10 days of the onset of COVID-19 symptoms (Rubio-Rivas et al., 2021). In another meta-analysis, in comparison to SOC or placebo, tocilizumab was found to reduce all-cause mortality, the requirement of MV, and the duration of hospitalization in COVID-19 patients (Piscoya et al., 2022). The results were similar to other meta-analyses as well (Boregowda et al., 2020; Khan et al., 2021; Rezaei et al., 2021; Tleyjeh et al., 2021; Zhang J. et al., 2022). The comparable efficacy and safety of baricitinib and tocilizumab in COVID-19 were reported in other network meta-analyses too (Kim et al., 2020; Siemieniuk et al., 2020; Zhang et al., 2021); however, the data required an update, particularly after the publication of latest large trials, such as the RECOVERY trial (Welcome Recovery Trial, 2022).

Baricitinib and tocilizumab are recommended to be used in a similar cohort of patients with COVID-19. We found that the efficacy and safety of both these drugs are comparable. The guidelines insist clinicians to consider factors, such as local guidance, ease of administration, access, storage (Agarwal et al., 2020; NICE 2022; Adults, Hospitalized Adults: Therapeutic Management, 2022), and cost of treatment (Lin et al., 2022) before selection of drugs. Baricitinib has a short half-life as compared to tocilizumab (12.5 h vs. 13 days) leading to a lesser chance of infection and long-term complications (Cantini et al., 2020). Also, it has a few drug-drug interactions (Conti et al., 2022). Unlike tocilizumab, baricitinib can be administered orally, can be stored easily, and is also much cheaper when used for a shorter duration. These factors qualify for its preferential use in low- or middle-income countries (Richardson and Stebbing, 2022).

The strengths of our study rest in the inclusion of all the latest studies, a large sample size of patients, and a robust analytical approach. Further, through the network meta-analysis, we have objectively demonstrated the comparative efficacy and safety of baricitinib and tocilizumab in hospitalized patients with COVID-19. There were a few limitations to the included studies. Some of the studies enrolled very few patients and these studies could have a high fragility index (Itaya et al., 2022). Although not planned as per the respective study protocols, some of the studies included crossover of treatment (baricitinib or tocilizumab with SOC) and administration of both baricitinib and tocilizumab in the same patients resulting in allocation bias. The comparator drug and the interval between symptom onset or diagnosis and enrolment varied across studies, which could have impacted the outcomes (Chen et al., 2021). Also, half of the included studies had a moderate risk of bias. Finally, although some studies included other immunomodulators apart from corticosteroids, we restricted the analyses to data on baricitinib or tocilizumab only. There were a few limitations to our review process as well. Data for all the outcomes of interest were unavailable, and hence, either could not be included in the review or could not be included in the pooled analysis (meta-analysis). The heterogeneity was large for some of the outcomes and the quality of evidence generated (GRADE) for some of the outcomes was low. Finally, in the absence of any head-on comparison trial between baricitinib and tocilizumab, the robustness of our results could be low.

In conclusion, we found out that treatment with baricitinib but not with tocilizumab led to a significant improvement in the 28-day mortality, as compared to that with the SOC. Treatment with baricitinib or tocilizumab led to a significant reduction in the duration of hospitalization and a significant improvement in the proportion of patients recovering clinically by day 28. From the safety point of view, both these drugs showed similar results. These two drugs are used almost interchangeably in hospitalized patients with COVID-19 who are already on systemic steroids, and hence, considering the better 28-day mortality data and other comparable efficacy and safety outcomes, baricitinib may be favored over tocilizumab because of the ease of its administration, shorter half-life, access, storage, and lower cost of treatment.

## Data Availability

The original contributions presented in the study are included in the article/Supplementary Material, further inquiries can be directed to the corresponding author.
