# Supplementary material for: Efficacy and safety of baricitinib and tocilizumab in hospitalized patients with COVID-19: A comparison using systematic review and meta-analysis
Source: Front Pharmacol. 2022 Oct 14;13:1004308. doi: 10.3389/fphar.2022.1004308 (PMC9624173; doi:10.3389/fphar.2022.1004308)
Supplement: Supplementary file 1 [file DataSheet1.docx]

***Supplementary Materials***

**Supplementary Table 1.** Literature search strategy.

| **Baricitinib** | | |
| --- | --- | --- |
| Patients | PubMed | COVID-19 OR (COVID-19 Virus Disease) OR (COVID-19 Virus Disease) OR (COVID-19 Virus) OR (Virus Disease, COVID-19) OR (COVID-19 Virus Infection) OR (Infection, COVID-19 Virus) OR (2019-nCoV Infection) OR (2019 nCoV Infection) OR (2019-nCoV Infections) OR (Infection, 2019-nCoV) OR (Coronavirus Disease-19) OR (Coronavirus Disease 2019) OR (2019 Novel Coronavirus Disease) OR (2019 Novel Coronavirus Infection) OR (2019-nCoV Disease) OR (2019 nCoV Disease) OR (2019-nCoV Diseases) OR (Disease, 2019-nCoV OR COVID19) OR (Coronavirus Disease 2019) OR (Disease 2019, Coronavirus) OR (SARS Coronavirus 2 Infection) OR (SARS-CoV-2 Infection) OR (Infection, SARS-CoV-2) OR (SARS CoV 2 Infection) OR (SARS-CoV-2 Infections) OR (COVID-19 Pandemic) OR (COVID-19 Pandemic) OR (COVID-19 Pandemics) OR (Pandemic, COVID-19) |
|  | Embase | ‘covid-19’ OR ‘covid-19’ OR ‘covid-19 virus disease’ OR ‘covid-19 virus disease’ OR ‘covid-19 virus diseases’ OR ‘disease, covid-19 virus’ OR ‘virus disease, covid-19’ OR ‘covid-19 virus infection’ OR ‘covid-19 virus infection’ OR ‘covid-19 virus infections’ OR ‘infection, covid-19 virus’ OR ‘virus infection, covid-19’ OR ‘2019-ncov infection’ OR ‘2019 ncov infection’ OR ‘2019-ncov infections’ OR ‘infection, 2019-ncov’ OR ‘coronavirus disease-19’ OR ‘coronavirus disease 19’ OR ‘2019 novel coronavirus disease’ OR ‘2019 novel coronavirus infection’ OR ‘2019-ncov disease’ OR ‘2019 ncov disease’ OR ‘2019-ncov diseases’ OR ‘disease, 2019-ncov’ OR ‘covid19’ OR ‘coronavirus disease 2019’ OR ‘disease 2019, coronavirus’ OR ‘sars coronavirus 2 infection’ OR ‘sars-cov-2 infection’ OR ‘infection, sars-cov-2’ OR ‘sars cov 2 infection’ OR ‘sars-cov-2 infections’ OR ‘covid-19 pandemic’ OR ‘covid 19 pandemic’ OR ‘covid-19 pandemics’ OR ‘pandemic, covid-19’ |
|  | *Clinical*[*Trial.gov*](http://trial.gov/) | Baricitinib AND COVID-19 |
| Intervention | PubMed | baricitinib OR Olumiant |
|  | Embase | baricitinib OR olumiant |
|  | *Clinical*[*Trial.gov*](http://trial.gov/) | Baricitinib AND COVID-19 |
| Tocilizumab | | |
| Patients | PubMed | COVID-19 OR (COVID-19 Virus Disease) OR (COVID-19 Virus Disease) OR (COVID-19 Virus) OR (Virus Disease, COVID-19) OR (COVID-19 Virus Infection) OR (Infection, COVID-19 Virus) OR (2019-nCoV Infection) OR (2019 nCoV Infection) OR (2019-nCoV Infections) OR (Infection, 2019-nCoV) OR (Coronavirus Disease-19) OR (Coronavirus Disease 2019) OR (2019 Novel Coronavirus Disease) OR (2019 Novel Coronavirus Infection) OR (2019-nCoV Disease) OR (2019 nCoV Disease) OR (2019-nCoV Diseases) OR (Disease, 2019-nCoV OR COVID19) OR (Coronavirus Disease 2019) OR (Disease 2019, Coronavirus) OR (SARS Coronavirus 2 Infection) OR (SARS-CoV-2 Infection) OR (Infection, SARS-CoV-2) OR (SARS CoV 2 Infection) OR (SARS-CoV-2 Infections) OR (COVID-19 Pandemic) OR (COVID-19 Pandemic) OR (COVID-19 Pandemics) OR (Pandemic, COVID-19) |
|  | Embase | ‘covid-19’ OR ‘covid-19’ OR ‘covid-19 virus disease’ OR ‘covid-19 virus disease’ OR ‘covid-19 virus diseases’ OR ‘disease, covid-19 virus’ OR ‘virus disease, covid-19’ OR ‘covid-19 virus infection’ OR ‘covid-19 virus infection’ OR ‘covid-19 virus infections’ OR ‘infection, covid-19 virus’ OR ‘virus infection, covid-19’ OR ‘2019-ncov infection’ OR ‘2019 ncov infection’ OR ‘2019-ncov infections’ OR ‘infection, 2019-ncov’ OR ‘coronavirus disease-19’ OR ‘coronavirus disease 19’ OR ‘2019 novel coronavirus disease’ OR ‘2019 novel coronavirus infection’ OR ‘2019-ncov disease’ OR ‘2019 ncov disease’ OR ‘2019-ncov diseases’ OR ‘disease, 2019-ncov’ OR ‘covid19’ OR ‘coronavirus disease 2019’ OR ‘disease 2019, coronavirus’ OR ‘sars coronavirus 2 infection’ OR ‘sars-cov-2 infection’ OR ‘infection, sars-cov-2’ OR ‘sars cov 2 infection’ OR ‘sars-cov-2 infections’ OR ‘covid-19 pandemic’ OR ‘covid 19 pandemic’ OR ‘covid-19 pandemics’ OR ‘pandemic, covid-19’ AND 'tocilizumab’ OR ‘actemra’ |
|  | *Clinical*[*Trial.gov*](http://trial.gov/) | Tocilizumab AND COVID-19 |
| Intervention | PubMed | tocilizumab OR Actemra |
|  | Embase | tocilizumab OR actemra |
|  | *Clinical*[*Trial.gov*](http://trial.gov/) | Tocilizumab AND COVID-19 |

**Supplementary Table 2.** GRADE tables representing the quality of generated evidence for the outcomes of baricitinib trials for which pooled analyses were performed.

| **Certainty assessment** | | | | | | | **№ of patients** | | **Effect** | | **Certainty** | **Importance** |
| --- | --- | --- | --- | --- | --- | --- | --- | --- | --- | --- | --- | --- |
| **№ of studies** | **Study design** | **Risk of bias** | **Inconsistency** | **Indirectness** | **Imprecision** | **Other considerations** | **Baricitinib** | **SOC** | **Relative (95% CI)** | **Absolute (95% CI)** |  |  |
| **28-day mortality** | | | | | | | | | | | | |
| 4 | randomised trials | serious^a^ | serious^b^ | not serious | not serious | none | 619/5478 (11.3%) | 712/5337 (13.3%) | **RR 0.69** (0.50 to 0.94) | **41 fewer per 1,000** (from 67 fewer to 8 fewer) | ⨁⨁◯◯ Low | CRITICAL |
| **Duration of hospitalization** | | | | | | | | | | | | |
| 4 | randomised trials | serious^a^ | not serious | not serious | not serious | none | 5478 | 5337 | - | MD **1.13 lower** (1.51 lower to 0.76 lower) | ⨁⨁⨁◯ Moderate | IMPORTANT |
| **Proportion of patients recovering clinically by day 28** | | | | | | | | | | | | |
| 3 | randomised trials | serious^c^ | not serious | not serious | not serious | none | 3789/4714 (80.4%) | 3556/4576 (77.7%) | **RR 1.24** (1.03 to 1.48) | **187 more per 1,000** (from 23 more to 373 more) | ⨁⨁⨁◯ Moderate | IMPORTANT |
| **Proportion of patients experiencing any serious adverse events** | | | | | | | | | | | | |
| 3 | randomised trials | not serious | not serious | not serious | not serious | none | 342/1330 (25.7%) | 408/1329 (30.7%) | **RR 0.76** (0.62 to 0.92) | **74 fewer per 1,000** (from 117 fewer to 25 fewer) | ⨁⨁⨁⨁ High | CRITICAL |
| **Proportion of patients experiencing serious infections** | | | | | | | | | | | | |
| 4 | randomised trials | serious^a^ | serious^b^ | not serious | not serious | none | 562/5478 (10.3%) | 572/5337 (10.7%) | **RR 0.86** (0.62 to 1.18) | **15 fewer per 1,000** (from 41 fewer to 19 more) | ⨁⨁◯◯ Low | IMPORTANT |
| **Proportion of patients experiencing cardiac serious adverse events** | | | | | | | | | | | | |
| 4 | randomised trials | serious^a^ | not serious | not serious | not serious | none | 108/5478 (2.0%) | 138/5337 (2.6%) | **RR 0.75** (0.58 to 0.97) | **6 fewer per 1,000** (from 11 fewer to 1 fewer) | ⨁⨁⨁◯ Moderate | IMPORTANT |
| **Proportion of patients experiencing venous thromboembolism** | | | | | | | | | | | | |
| 4 | randomised trials | serious^a^ | not serious | not serious | not serious | none | 228/5478 (4.2%) | 221/5337 (4.1%) | **RR 1.00** (0.83 to 1.21) | **0 fewer per 1,000** (from 7 fewer to 9 more) | ⨁⨁⨁◯ Moderate | IMPORTANT |

**CI:** confidence interval; **MD:** mean difference; **RR:** risk ratio

#### Explanations

a. Half of the studies had a moderate risk of bias.

b. The unexplained heterogeneity was high.

c. Two studies had a moderate risk of bias.

**Supplementary Table 3.** GRADE tables representing the quality of generated evidence for the outcomes of tocilizumab trials for which pooled analyses were performed.

| **Certainty assessment** | | | | | | | **№ of patients** | | **Effect** | | **Certainty** | **Importance** |
| --- | --- | --- | --- | --- | --- | --- | --- | --- | --- | --- | --- | --- |
| **№ of studies** | **Study design** | **Risk of bias** | **Inconsistency** | **Indirectness** | **Imprecision** | **Other considerations** | **Tocilizumab** | **SOC** | **Relative (95% CI)** | **Absolute (95% CI)** |  |  |
| **28-day mortality** | | | | | | | | | | | | |
| 16 | randomised trials | serious^a^ | not serious | not serious | not serious | none | 882/4073 (21.7%) | 928/3541 (26.2%) | **RR 0.87** (0.71 to 1.07) | **34 fewer per 1,000** (from 76 fewer to 18 more) | ⨁⨁⨁◯ Moderate | CRITICAL |
| **14-day mortality** | | | | | | | | | | | | |
| 7 | randomised trials | not serious | not serious | not serious | not serious | none | 51/717 (7.1%) | 40/641 (6.2%) | **RR 1.19** (0.66 to 2.13) | **12 more per 1,000** (from 21 fewer to 71 more) | ⨁⨁⨁⨁ High | IMPORTANT |
| **Duration of hospitalization** | | | | | | | | | | | | |
| 9 | randomised trials | serious^b^ | serious^c^ | not serious | not serious | none | 3194 | 2915 | - | MD **2.8 lower** (4.17 lower to 1.43 lower) | ⨁⨁◯◯ Low | IMPORTANT |
| **Proportion of patients requiring mechanical ventilation by day 28** | | | | | | | | | | | | |
| 11 | randomised trials | serious^d^ | not serious | not serious | not serious | none | 961/3855 (24.9%) | 1031/3327 (31.0%) | **RR 0.79** (0.71 to 0.88) | **65 fewer per 1,000** (from 90 fewer to 37 fewer) | ⨁⨁⨁◯ Moderate | IMPORTANT |
| **Proportion of patients requiring ICU admission by day 28** | | | | | | | | | | | | |
| 7 | randomised trials | not serious | not serious | not serious | not serious | none | 174/909 (19.1%) | 162/664 (24.4%) | **RR 0.83** (0.57 to 1.19) | **41 fewer per 1,000** (from 105 fewer to 46 more) | ⨁⨁⨁⨁ High | IMPORTANT |
| **Duration of ICU stay by day 28** | | | | | | | | | | | | |
| 4 | randomised trials | serious^e^ | not serious | not serious | not serious | none | 424 | 450 | - | MD **4.25 lower** (9.39 lower to 0.89 lower) | ⨁⨁⨁◯ Moderate | IMPORTANT |
| **Duration of ventilator-free days by day 28** | | | | | | | | | | | | |
| 5 | randomised trials | not serious | not serious | not serious | not serious | none | 528 | 372 | - | MD **3.29 higher** (0.61 lower to 7.19 higher) | ⨁⨁⨁⨁ High | IMPORTANT |
| **Proportion of patients recovering clinically by day 28** | | | | | | | | | | | | |
| 10 | randomised trials | serious^e^ | not serious | not serious | not serious | none | 2003/3131 (64.0%) | 1610/2878 (55.9%) | **RR 1.41** (1.12 to 1.78) | **229 more per 1,000** (from 67 more to 436 more) | ⨁⨁⨁◯ Moderate | IMPORTANT |
| **Proportion of patients experiencing any serious adverse events** | | | | | | | | | | | | |
| 14 | randomised trials | not serious | not serious | not serious | not serious | none | 262/2353 (11.1%) | 229/1823 (12.6%) | **RR 0.68** (0.48 to 1.00) | **40 fewer per 1,000** (from 65 fewer to 0 fewer) | ⨁⨁⨁⨁ High | CRITICAL |
| **Proportion of patients experiencing cardiac SAE** | | | | | | | | | | | | |
| 14 | randomised trials | not serious | not serious | not serious | not serious | none | 133/4089 (3.3%) | 161/3754 (4.3%) | **RR 0.82** (0.64 to 1.04) | **8 fewer per 1,000** (from 15 fewer to 2 more) | ⨁⨁⨁⨁ High | IMPORTANT |
| **Proportion of patients experiencing venous thromboembolism** | | | | | | | | | | | | |
| 7 | randomised trials | not serious | not serious | not serious | not serious | none | 19/1107 (1.7%) | 30/1077 (2.8%) | **RR 0.59** (0.22 to 1.55) | **11 fewer per 1,000** (from 22 fewer to 15 more) | ⨁⨁⨁⨁ High | IMPORTANT |
| **Proportion of patients experiencing serious bleeding** | | | | | | | | | | | | |
| 7 | randomised trials | not serious | not serious | not serious | not serious | none | 37/1629 (2.3%) | 22/1222 (1.8%) | **RR 0.09** (0.63 to 1.87) | **16 fewer per 1,000** (from 7 fewer to 16 more) | ⨁⨁⨁⨁ High | IMPORTANT |

**CI:** confidence interval; **MD:** mean difference; **RR:** risk ratio

#### Explanations

a. Eight studies had a moderate risk of bias.

b. Five studies had a moderate risk of bias.

c. The unexplained heterogeneity was high.

d. Six studies had a moderate risk of bias.

e. Half of the studies had a moderate risk of bias.

| **Author, year** | **Bias arising from the randomization process** | **Bias due to deviations from intended interventions** | **Bias due to missing outcome data** | **Bias due to measurement of the outcome** | **Bias in selection of the reported result** | **Overall bias** |
| --- | --- | --- | --- | --- | --- | --- |
| Horby, et al., 2022 |  |  |  |  |  |  |
| Kalil, et al., 2020 |  |  |  |  |  |  |
| Marconi, et al., 2021 |  |  |  |  |  |  |
| Wesley Ely, et al., 2022 |  |  |  |  |  |  |
| Abani, et al., 2021 |  |  |  |  |  |  |
| Broman, et al., 2022 |  |  |  |  |  |  |
| Declercq, et al., 2021 |  |  |  |  |  |  |
| Gordon, et al., 2021 |  |  |  |  |  |  |
| Hermine, et al., 2021 |  |  |  |  |  |  |
| Hermine, et al., 2022a |  |  |  |  |  |  |
| Hermine, et al., 2022b |  |  |  |  |  |  |
| Naik, et al., 2021 |  |  |  |  |  |  |
| Rosas, et al., 2021a |  |  |  |  |  |  |
| Rosas, et al., 2021b |  |  |  |  |  |  |
| Rutgers, et al., 2021 |  |  |  |  |  |  |
| Salama, et al., 2021 |  |  |  |  |  |  |
| Salvarani, et al., 2021 |  |  |  |  |  |  |
| Soin, et al., 2021 |  |  |  |  |  |  |
| Stone, et al., 2020 |  |  |  |  |  |  |
| Veiga, et al., 2021 |  |  |  |  |  |  |
| Wang, et al., 2021 |  |  |  |  |  |  |
| Zhao, et al., 2021 |  |  |  |  |  |  |

| **Index** | |
| --- | --- |
|  | Low risk of bias |
|  | Moderate risk of bias |
|  | High risk of bias |

**Supplementary Figure 1.** Risk of bias assessment following the revised Cochrane risk-of-bias 2 tool.

**
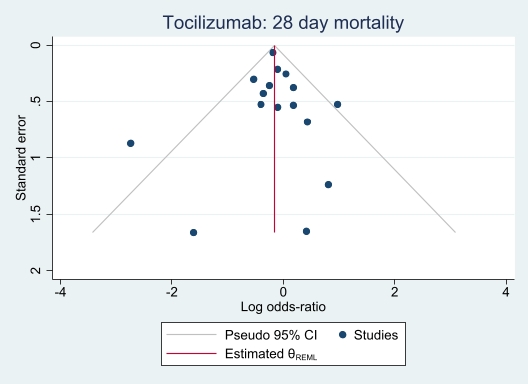
**

**Supplementary Figure 2.** Funnel plot showing the effect of tocilizumab on the 28-day mortality.

**
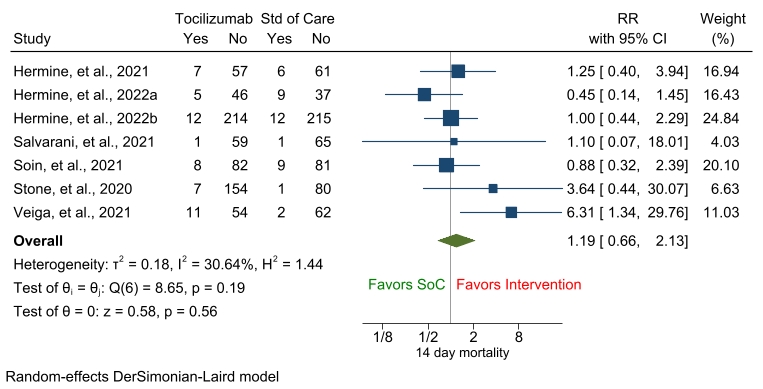
**

**Supplementary Figure 3.** 14-day mortality following treatment with tocilizumab as compared to that with the standard of care (SOC).


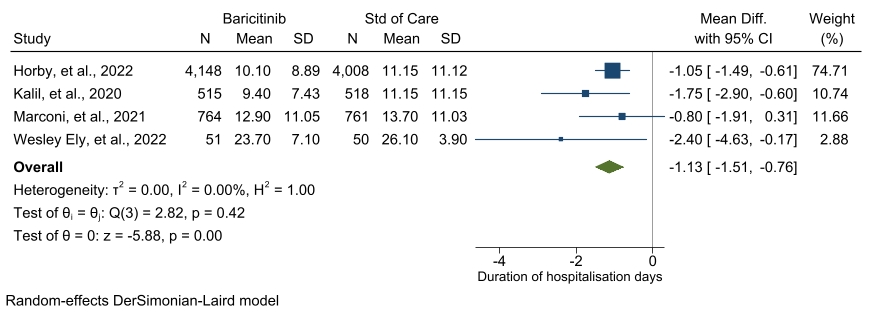


.

**Supplementary Figure 4.** Duration of hospitalization (days) following treatment with baricitinib as compared to that with the standard of care (SOC).


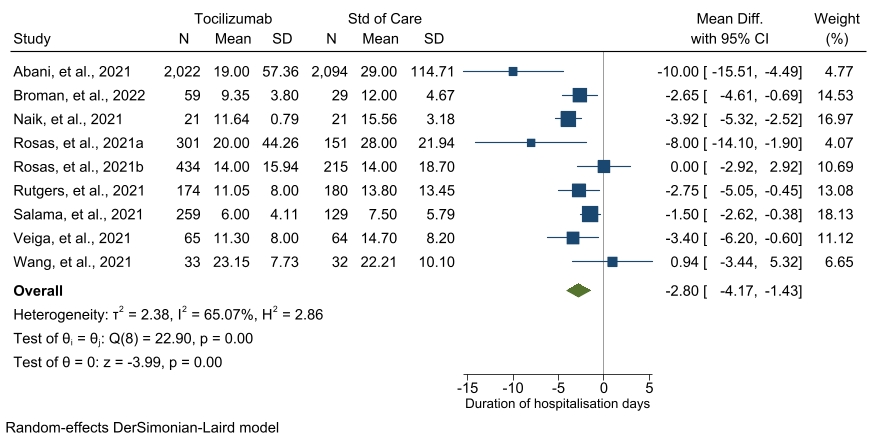


**Supplementary Figure 5.** Duration of hospitalization (days) following treatment with tocilizumab as compared to that with the standard of care (SOC).


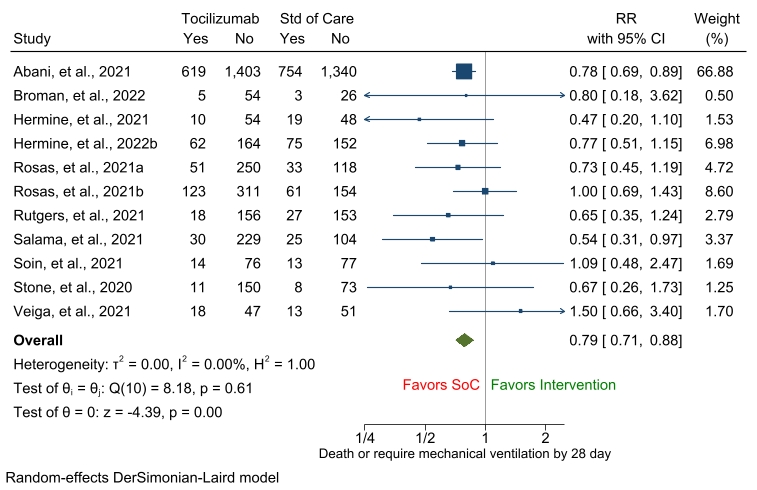


**Supplementary Figure 6.** Proportion of patients requiring mechanical ventilation by day 28 following treatment with tocilizumab as compared to that following treatment with the standard of care (SOC).


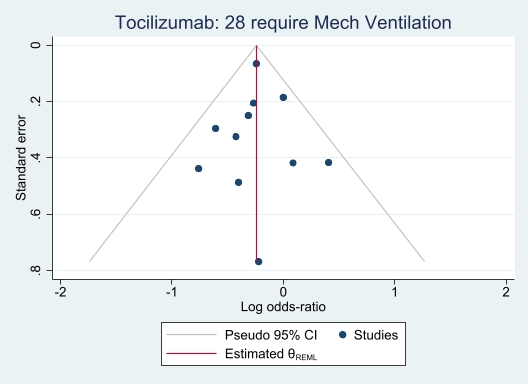


**Supplementary Figure 7.** Funnel plot showing the effect of tocilizumab on the proportion of patients requiring mechanical ventilation by day 28.


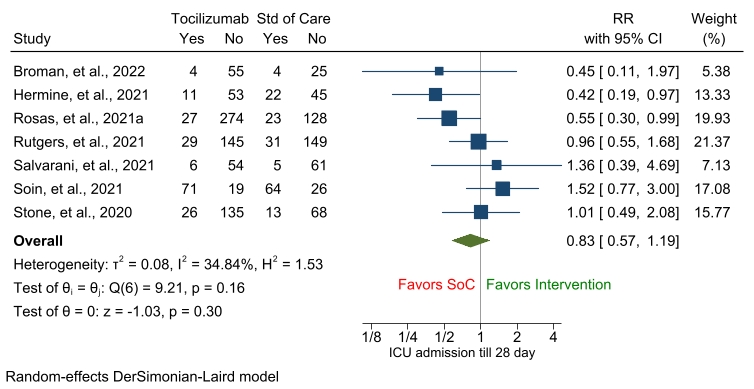


**Supplementary Figure 8.** Proportion of patients requiring ICU admission by day 28 following treatment with tocilizumab as compared to that with the standard of care (SOC).


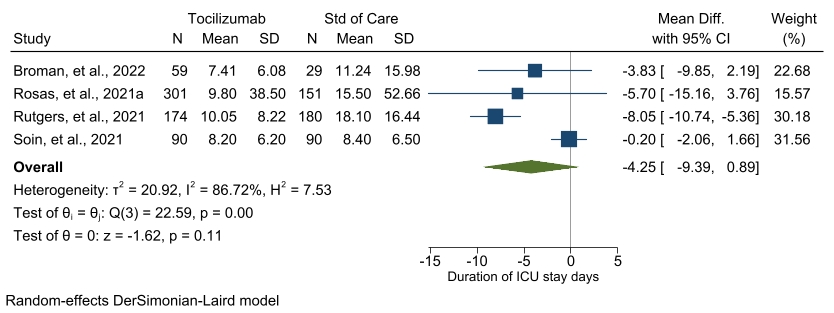


**Supplementary Figure 9.** Duration of intensive care unit stay (days) by day 28 following treatment with tocilizumab as compared to that with the standard of care (SOC).


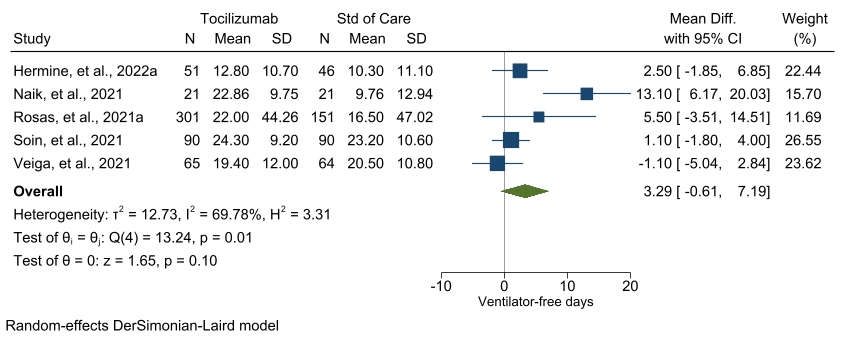


**Supplementary Figure 10.** Duration of ventilator-free days (days) by day 28 following treatment with tocilizumab as compared to that with the standard of care (SOC).


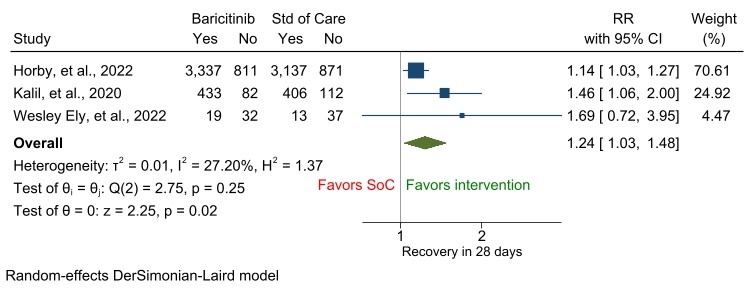


**Supplementary Figure 11.** Proportion of patients recovering clinically by day 28 following treatment with baricitinib as compared to that with the standard of care (SOC).


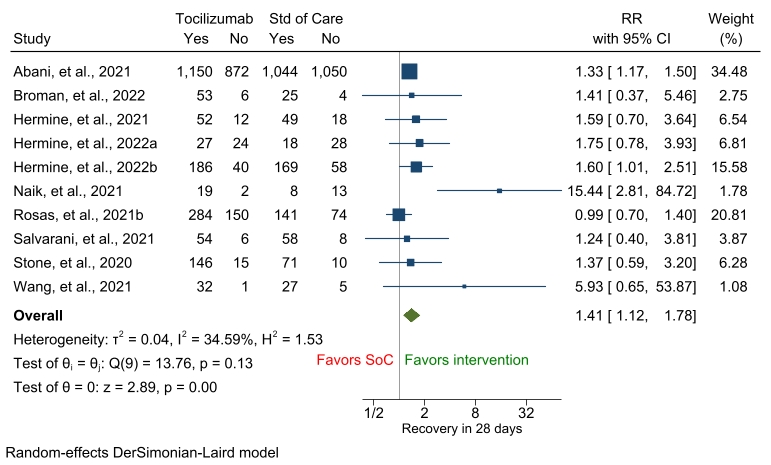


**Supplementary Figure 12.** Proportion of patients recovering clinically by day 28 following treatment with tocilizumab as compared to that following treatment with the standard of care (SOC).


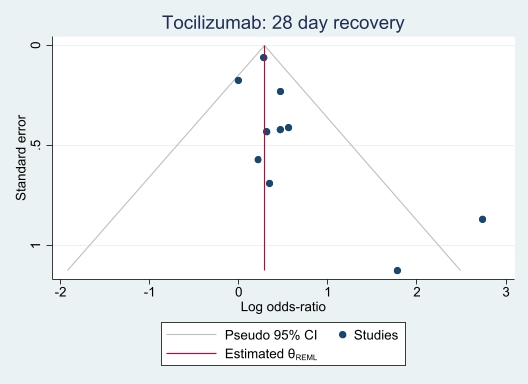


**Supplementary Figure 13.** Funnel plot showing the effect of tocilizumab on the proportion of patients recovering clinically by day 28.


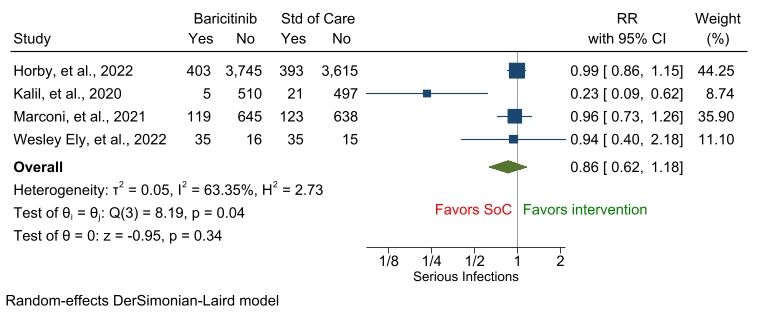


**Supplementary Figure 14.** Proportion of patients experiencing serious infections following treatment with baricitinib as compared to that with the standard of care (SOC).


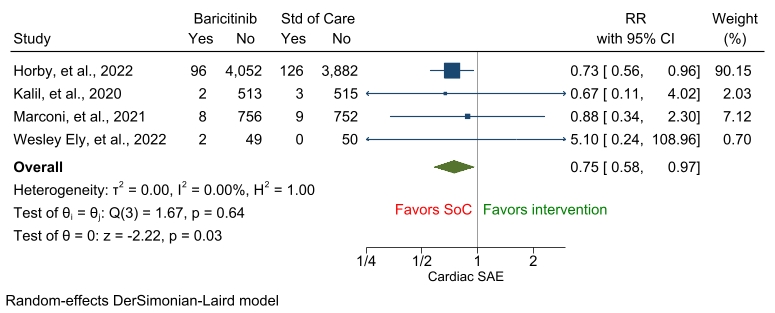


**Supplementary Figure 15.** Proportion of patients experiencing cardiac SAEs following treatment with baricitinib as compared to that with the standard of care (SOC).

**
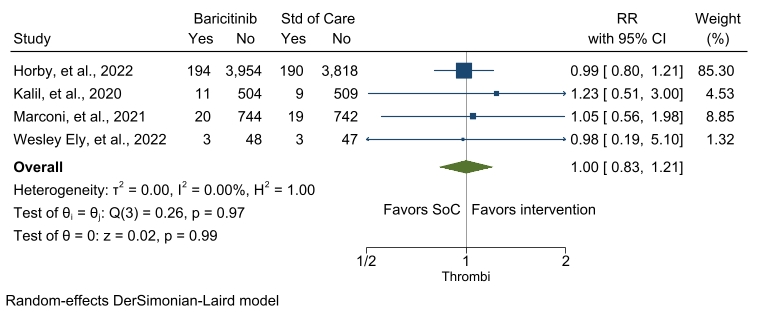
**

**Supplementary Figure 16.** Proportion of patients experiencing venous thromboembolism following treatment with baricitinib as compared to that with the standard of care (SOC).


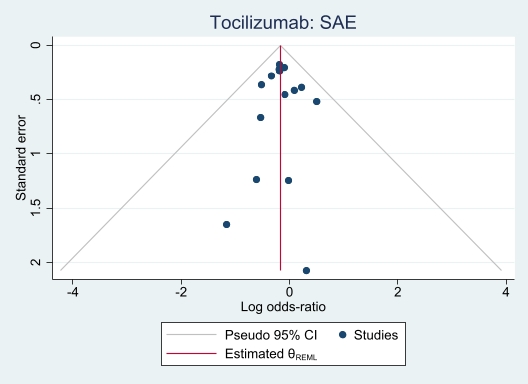


**Supplementary Figure 17.** Funnel plot showing the effect of tocilizumab on the proportion of patients experiencing any serious adverse events.


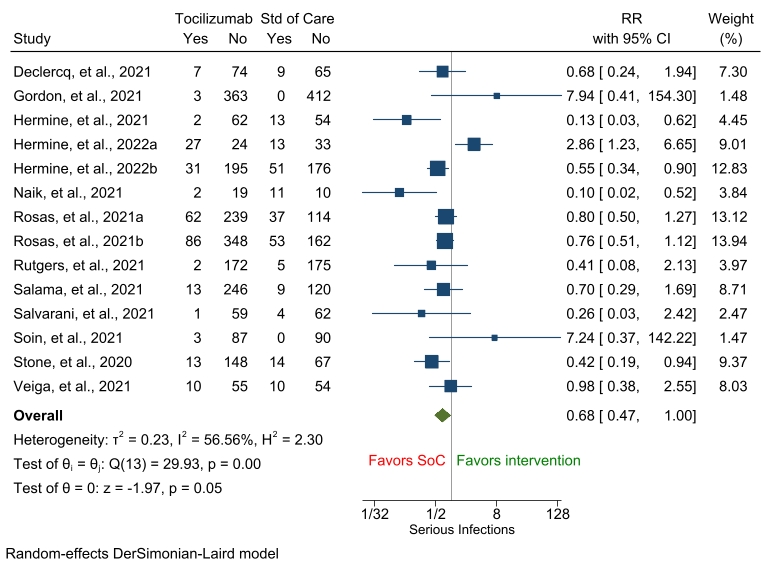


**Supplementary Figure 18.** Proportion of patients experiencing serious infections following treatment with tocilizumab as compared to that with the standard of care (SOC).


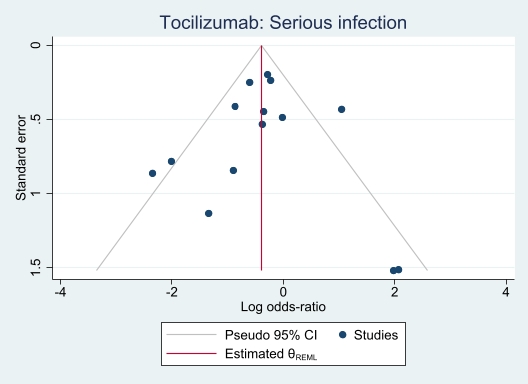


**Supplementary Figure 19.** Funnel plot showing the effect of tocilizumab on the proportion of patients experiencing serious infections.


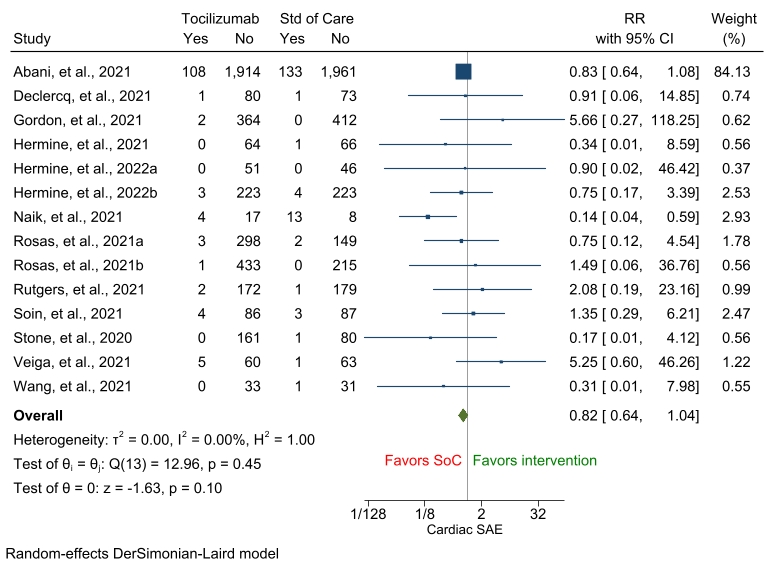


**Supplementary Figure 20.** Proportion of patients experiencing cardiac SAE following treatment with tocilizumab as compared to that with the standard of care (SOC).


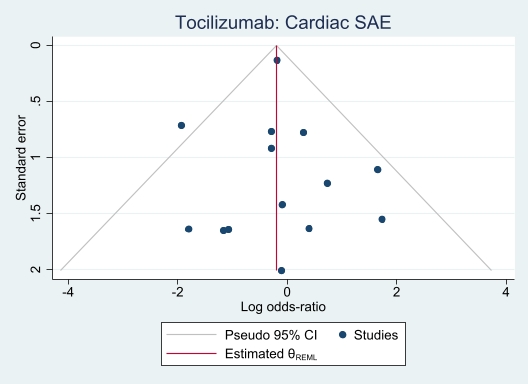


**Supplementary Figure 21.** Funnel plot showing the effect of tocilizumab on the proportion of patients experiencing cardiac SAE.


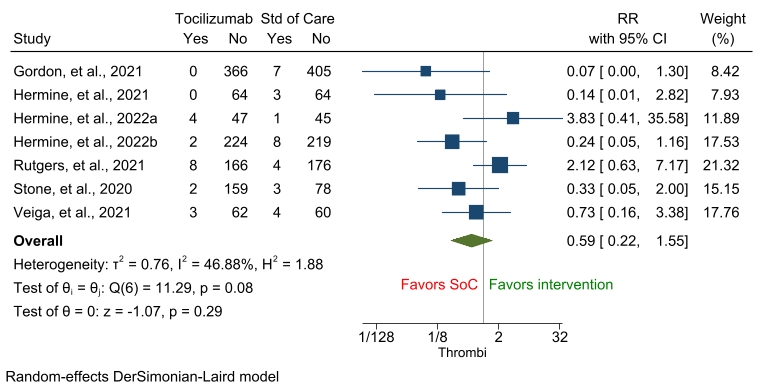


**Supplementary Figure 22.** Proportion of patients experiencing venous thromboembolism following treatment with tocilizumab as compared to that with the standard of care (SOC).


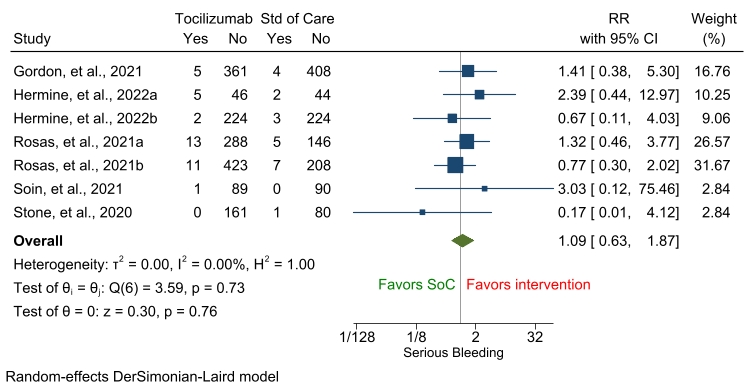


**Supplementary Figure 23.** Proportion of patients experiencing serious bleeding following treatment with tocilizumab as compared to that with the standard of care (SOC).


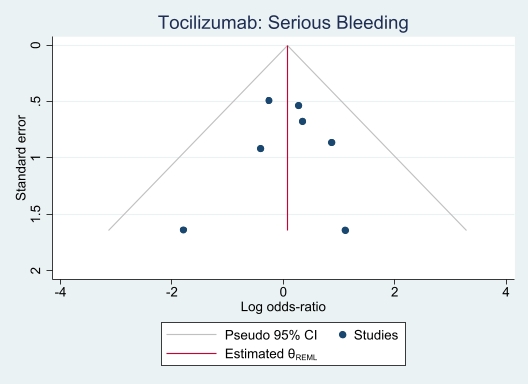


**Supplementary Figure 24.** Funnel plot showing the effect of tocilizumab on the proportion of patients experiencing serious bleeding.


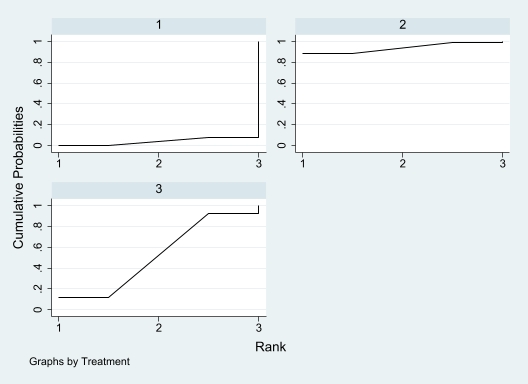


**Supplementary Figure 25.** Rankogram and cumulative ranking of the 28-day mortality in the network meta-analysis (1, baricitinib; 2, tocilizumab; and 3, standard of care).


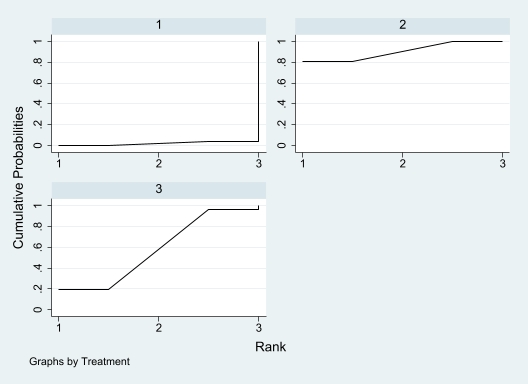


**Supplementary Figure 26.** Rankogram and cumulative ranking of the serious adverse events in the network meta-analysis (1, baricitinib; 2, tocilizumab; and 3, standard of care).


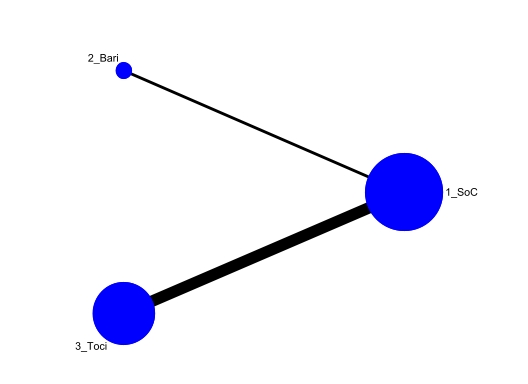


**Supplementary Figure 27.** Network map of the comparison of the 28-day mortality between baricitinib, tocilizumab, and the standard of care (SOC). The nodes were weighed by the number of included studies in each treatment comparison, while the edges were weighed by the sample size of that comparison.


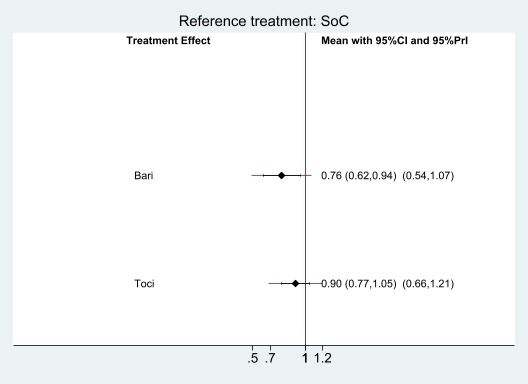


**Supplementary Figure 28.** Interval plot of the comparison of the 28-day mortality between baricitinib, tocilizumab, and the standard of care (SOC).


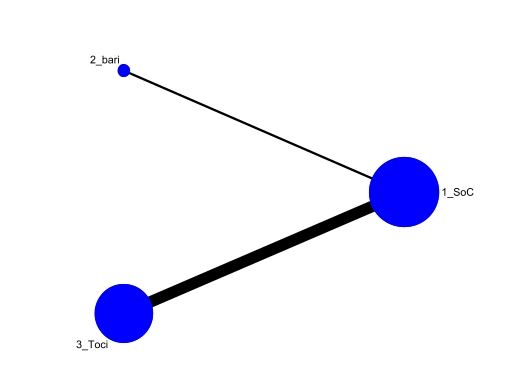


**Supplementary Figure 29.** Network map of the comparison of all serious adverse events between baricitinib, tocilizumab, and the standard of care (SOC). The nodes were weighed by the number of included studies in each treatment comparison, while the edges were weighed by the sample size of that comparison.


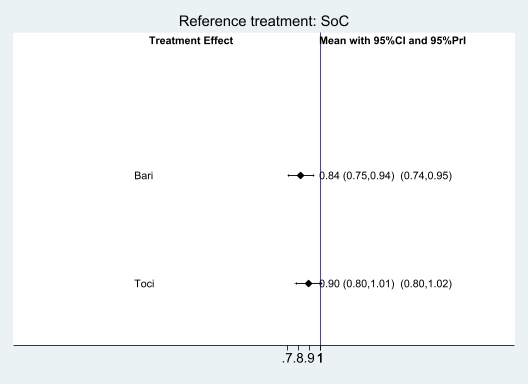


**Supplementary Figure 30.** Interval plot of the comparison of all serious adverse events between baricitinib, tocilizumab, and the standard of care (SOC).
